# Probiotics alleviate constipation and inflammation in late gestating and lactating sows

**DOI:** 10.1038/s41522-023-00434-z

**Published:** 2023-09-23

**Authors:** Teng Ma, Weiqiang Huang, Yalin Li, Hao Jin, Lai-Yu Kwok, Zhihong Sun, Heping Zhang

**Affiliations:** 1 Inner Mongolia Key Laboratory of Dairy Biotechnology and Engineering, Inner Mongolia Agricultural University, Hohhot, Inner Mongolia China; 2 Key Laboratory of Dairy Products Processing, Ministry of Agriculture and Rural Affairs, Inner Mongolia Agricultural University, Hohhot, Inner Mongolia China; 3 Key Laboratory of Dairy Biotechnology and Engineering, Ministry of Education, Inner Mongolia Agricultural University, Hohhot, Inner Mongolia China

**Keywords:** Microbiome, Next-generation sequencing, Metagenomics, Microbial genetics, Policy and public health in microbiology

## Abstract

Constipation and systemic inflammation are common in late pregnant and lactating sows, which cause health problems like uteritis, mastitis, dystocia, or even stillbirth, further influencing piglets’ survival and growth. Probiotic supplementation can improve such issues, but the beneficial mechanism of relieving constipation and enhancing gut motility remains underexplored. This study aimed to investigate the effects and mechanism of probiotic supplementation in drinking water to late pregnant sows on constipation, inflammation, and piglets’ growth performance. Seventy-four sows were randomly allocated to probiotic (*n* = 36) and control (*n* = 38) groups. Probiotic treatment significantly relieved sow constipation, enhanced serum IL-4 and IL-10 levels while reducing serum IL-1β, IL-12p40, and TNF-α levels, and increased piglet daily gain and weaning weight. Furthermore, probiotic administration reshaped the sow gut bacteriome and phageome structure/diversity, accompanied by increases in some potentially beneficial bacteria. At 113 days of gestation, the probiotic group was enriched in several gut microbial bioactive metabolites, multiple carbohydrate-active enzymes that degrade pectin and starch, fecal butyrate and acetate, and some serum metabolites involved in vitamin and amino acid metabolism. Our integrated correlation network analysis revealed that the alleviation of constipation and inflammation was associated with changes in the sow gut bacteriome, phageome, bioactive metabolic potential, and metabolism.

## Introduction

Gestation, lactation, and newborn periods are the core stages for management in the large-scale production of sows and piglets^1^. From gestation to and during lactation, sows undergo dramatic changes in their physiology, metabolism, and immunity to meet the nutritional and energy requirements^2^. However, imbalanced nutrition during pregnancy and lactation not only causes a range of health issues in sows, including constipation, abortion, and intrauterine growth retardation but also increases the risk of problems like low weaning weight and high rates of diarrhea in offspring^3^. Therefore, reducing inflammation, maintaining a healthy metabolism, and relieving constipation during late gestation and lactation of sows are extremely important in ensuring sow reproductive performance and the growth of offspring^4,5^. Various efforts have been put to improve the health of gestating sows. For example, the supplementation of dietary fiber and L-glutamine to relieve constipation in gestation sows, but inferences drawn from different studies are largely inconsistent^6,7^. Antibiotics have been used in the swine industry to reduce inflammation, but the potential risks to human health, spread of antibiotic resistance, and disruption of gut microbiota homeostasis are alarming concerns^8^. The gut microbiota plays an important role in maintaining gut homeostasis, acquiring and assimilating nutrients, regulating inflammation, and guarding against pathogens in both sows and piglets^9,10^. The metabolites produced by the sow gut microbiota can be transferred to the offspring, promoting the maturation and development of the immune system in newborn piglets^11,12^.

In the past few years, the swine industry has benefited from probiotic research as it is increasingly recognized that, when applied in pig farming, probiotics can serve as health and/or growth promoters by improving the gut microbiome and the bioactive metabolome of the animals^13^. Probiotics have been evaluated in sows for their beneficial effects on their well-being, health promotion, and reproductive performance^14,15^. Supplementing probiotics to gestating sows during late pregnancy or lactation stages has been shown to exert desirable effects both on the sows and the newborns, e.g., improving colostrum quality, shortening estrus intervals, relieving constipation, reducing serum inflammatory factors, increasing piglet weaning weight, regulating host immunity, and reducing diarrhea rate and mortality^16^. Moreover, other studies showed that probiotic supplementation could modulate the gut microbiota diversity and community structure in gestating sows and piglets, meanwhile reducing the abundance of pathogenic bacteria, such as Salmonella, Clostridium, and Escherichia coli^17,18^. However, the underlying probiotic mechanism is not well understood, particularly how the gut microbes and their metabolites alleviate constipation and inflammation during late gestation.

On the other, one problem in this field of research is that the reported outcomes often vary between studies. Some trials observed no statistically significant benefits after probiotic administration. Indeed, the efficacy of probiotics depends on many factors, including the physiological status and age of sows, farm environment, diet composition, probiotic intervention route, strain combination, and dose^19^. In addition, compound probiotics may have a potentially higher efficacy due to a greater extent of synergism and symbiosis between the probiotics or interaction with the host gut microbial community^20^. Owing to the variability in one or multiple aforementioned factors, it has not been easy to directly compare results reported by different studies or between laboratories. Since the probiotic function is both strain- and host-specific, it is still necessary to conduct extensive and high-quality basic research to expand the diversity of candidate strains, assessing their stability and consistency, and further elucidating the beneficial mechanisms. The probiotic strains, Bifidobacterium lactis Probio-M8 (Probio-M8) and Lacticaseibacillus rhamnosus Probio-M9 (Probio-M9), were originally isolated from human breast milk; and a previous integrated culture-dependent/-independent study found that Probio-M8 could translocate to the infant’s intestine via oral‑/entero‑mammary routes^21^. These strains have previously been reported to relieve constipation and gastrointestinal symptoms in Parkinson’s patients^22^, reduce body inflammation and relieve acute respiratory tract infections in young children^23^, and increase antitumor immune efficacy by combining/not combining anti-PD-1^24,25^. Moreover, complex probiotics containing both strains have been used in multiple animal and clinical trials^26,27^, demonstrating their added beneficial effects when used in adjunct to conventional drugs for managing ulcerative colitis and irritable bowel syndrome^28,29^.

This study hypothesized that compound probiotics could improve inflammation and constipation in late-gestating and lactating sows by modulating the gut microbiota and blood metabolites. Therefore, the first objective of this longitudinal trial was to evaluate the anti-inflammatory effect and immune responses of supplementing complex probiotics (comprised of Probio-M8 and Probio-M9) to sows during late gestation and lactation. The second objective of this study was to further dissect the observed beneficial mechanisms by identifying probiotic-driven changes in the gut microbiota and blood metabolite biomarkers. This work provides scientific knowledge and practical information on the use of probiotics in improving pig farming performance and efficiency, expanding the scope of probiotic application in animals at late gestation and lactation.

## Results

### Viable probiotic bacterial counts in the probiotic powder, pre-solution, diluted pre-solution, and drinking water system

The number of viable probiotic bacteria in the probiotic powder, pre-solution, diluted pre-solution, and samples collected in the drinking water system was monitored during the trial (Supplementary Table 1). As expected, the probiotic powders contained both Probio-M8 and Probio-M9 (5.70 ± 0.23 × 10^10^ CFU/g and 5.20 ± 0.48 × 10^10^ CFU/g, respectively). The pre-solution was prepared in a ratio of 1: 100 (probiotic powder: water), and its viable counts of Probio-M8 and Probio-M9 were 6.03 ± 0.47 × 10^8^ CFU/g and 3.44 ± 0.35 × 10^8^ CFU/mL, respectively. We also monitored the hourly changes in the number of viable bacteria in the pre-solution for three hours after the 100-fold dilution. The viability of the two probiotic strains was stable, with viable counts ranging from 4.55 ± 0.43 to 5.73 ± 0.5 × 10^8^ CFU/mL for Probio-M8 and 3.11 ± 0.18 to 3.56 ± 0.14 × 10^8^ CFU/mL for Probio-M9. The probiotic-containing drinking water was indeed a 200-fold diluted pre-solution, and its probiotic viability was also checked hourly for three hours after the dilution. The viable counts were consistently higher than 10^6^ CFU/mL for both strains. The two probiotic strains were not detected in the drinking water system of the control group, confirming that there was no cross-contamination between the drinking water systems of the two groups.

### Probiotics administration during late gestation and lactation alleviated constipation and improved piglet performance

**Fig. 1:**
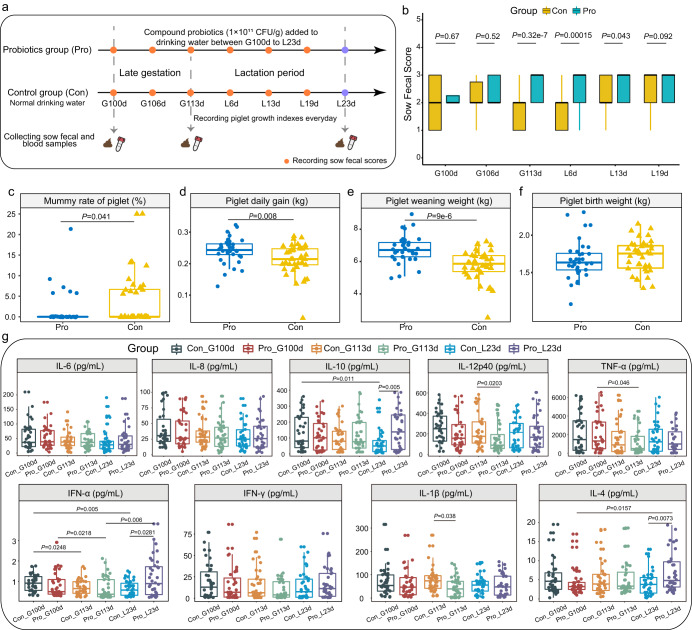
Trial design and the effects of probiotics on constipation, reproductive and piglet growth performance, and serum cytokine profile. **a** Intervention measures and sample collection from late gestation (100 days of gestation, G100d) to lactation (23 days of lactation, L23d). G113d represents 113 days of gestation, while L6d, L13d, and L19d represent 6, 13, and 19 days of lactation, respectively. **b** Comparison of sow fecal scores between probiotic (Pro) and control (Con) groups from late gestation to lactation. The sow fecal score is inversely related to the severity of constipation. Comparison of (**c**) mummy rate, (**d**) daily gain, (**e**) weaning weight, and (**f**) birth weight of piglets between two groups. **g** Changes in serum cytokine levels at different time points. Data were presented as means ± SEM. Differences in serum concentrations of interleukin-6 (IL-6), interleukin-8 (IL-8), interleukin-10 (IL-10), interleukin-12p40 (IL-12p40), tumoral necrosis factor-alpha (TNF-α), interferon-α (IFN-α), interferon-γ (IFN-γ), interleukin-1β (IL-1β), and interleukin-4 (IL-4) between groups were evaluated using Wilcoxon tests. The center line of the box plot represents the median, the bounds of box represent IQR, and whiskers represent data other than the upper and lower quartiles.

No significant difference was found in the average daily feed intake or water consumption between probiotic and control sows during late gestation and lactation (average daily feed intake: 2.7 kg and 2.75 kg in late gestation, 5.34 kg and 5.41 kg in lactation period, in probiotic and control groups, respectively; average daily water intake: 12.86 kg and 12.31 kg in late gestation, 48.58 kg and 48.39 kg in lactation period, in probiotic and control groups, respectively; *P* > 0.05 in all cases; Supplementary Table 2). Then, the effect of probiotic consumption on alleviating constipation in sows in late gestation (corresponding to 100, 106, and 113 days of gestation, represented by G100d, G106d, and G113d, respectively; six, 13, and 19 days of lactation, represented by L6d, L13d, and L19d, respectively) was evaluated by the sow fecal score, which was inversely related with the severity of constipation (Fig. 1a). Compared with the control group, probiotics administration significantly reduced the severity of constipation of sows in late gestation (G113d, *P* < 0.001; Wilcoxon test) and lactation (L6d, *P* < 0.001; L13d, *P* = 0.043; Wilcoxon test; Fig. 1b; Supplementary Fig. 1a). Then, the effects of compound probiotics in drinking water on the reproductive performance of sows were evaluated. No significant difference was detected in the total litter size, healthy litter rate, weak litter rate, and stillbirth rate between probiotic and control groups (*P* > 0.05; Supplementary Table. 3). However, the mummification rate of piglets in the probiotic group was significantly lower than in the control group (*P* < 0.05; Wilcoxon test; Fig. 1c), suggesting that supplementation of compound probiotics may have beneficial effects on the sow reproductive performance.

All piglets were vaginally delivered, and the birth weight, daily weight gain, weaning weight, and diarrhea rate of piglets were monitored to evaluate the effect of probiotic administration on piglet performance. The daily weight gain and weaning weight of piglets were significantly higher in the probiotic group compared with the control group (*P* < 0.001; Wilcoxon test; Figs. 1d, 1e), but no significance was found in the birth weight between the two groups (Fig. 1f). In addition, the number of diarrhea days of piglets was non-significantly lower in the probiotic group compared with the control group (*P* > 0.05; Wilcoxon test). These data indicated that probiotic intake not only effectively alleviated constipation in sows at late gestation and lactation but improved the growth performance of piglets.

### Probiotics administration modulated the serum cytokine profile in late gestating and lactating sows

The host immune responses could affect the gut inflammatory state and disrupt the intestinal barrier, leading to general and gastrointestinal health issues in pregnant and lactating sows. The serum cytokine profile of sows might reflect their inflammatory state. Therefore, to reveal the immunomodulatory effect of supplementing compound probiotics in drinking water to late gestating and lactating sows, their serum levels of pro-inflammatory (such as interleukin [IL]-1β, IL-12p40, tumor necrosis factor [TNF]-α, interferon [IFN]-γ, IL-6. and IL-8), anti-inflammatory (such as IL-4 and IL-10), and pleiomorphic (such as IFN-α) cytokines were monitored. Compared with the control group, the serum levels of some of the monitored cytokines (IL-1β and IL-12p40) were significantly lower in the probiotic group at G113d (*P* < 0.05; Wilcoxon test). In contrast, the serum levels of IFN-α, IL-4, and IL-10 in the probiotic group were significantly higher at L23d (*P* < 0.05; Wilcoxon test). Moreover, the serum TNF-α levels of sows decreased only in the probiotic group but not in the control group at G113d (*P* < 0.05; Wilcoxon test). Compared with G100d, the serum levels of IFN-α of sows in both groups were significantly lower at G113d (*P* < 0.05; Wilcoxon test). There were no significant differences in other serum cytokines (IFN-γ, IL-6, and IL-8) between the two groups at all three monitored time points (G100d, G113d, and L23d; Fig. 1g). We also identified the daily gain and weaning weight of piglets had a significant positive correlation with the serum IFN-α level of sows (*r* > 0.4) while exhibiting a significant negative correlation with IL-8 (*r* < −0.4). In contrast, the serum IL-4 level showed a significant positive correlation with piglet daily gain (*r* > 0.4; Supplementary Fig. 1b). These results highlight the role of probiotics in reducing systemic inflammation in late gestation and lactation sows.

### Probiotic supplementation modulated the gut microbiota composition of sows in late gestation and lactation

**Fig. 2:**
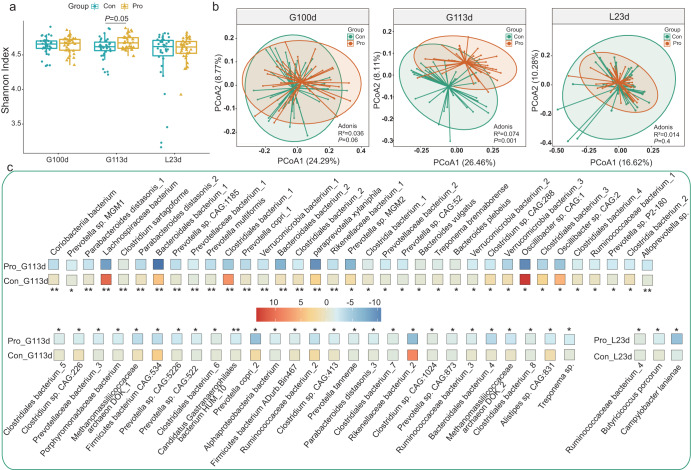
Microbial diversity and species-level genome bins (SGBs) features in sow gut microbiota. **a** Shannon diversity index and (**b**) principal coordinates analysis (PCoA) score plots of probiotic (Pro) and control (Con) groups at days 100 days of gestation (G100d), 113 days of gestation (G113d), and 23 days of lactation (L23d). Symbols representing samples of the two groups are shown in different colors. Results of Adonis tests are shown at the right lower corner of PCoA plots. **c** Responsive SGBs showing significant differential abundance between two groups at different time points. The color scale illustrates the relative abundance; the intensity of red and blue represent higher and lower relative abundances, respectively. Wilcoxon test was used to evaluate statistical differences; *P* values were corrected by the Benjamini-Hochberg procedure; *P < 0.05; **P < 0.01. Data were expressed as the means ± SEM. The center line of the box plot represents the median, the bounds of box represent IQR, and whiskers represent data other than the upper and lower quartiles.

The fecal microbiota of the sows was analyzed at three-time points (G100d, G113d, and L23d) by shotgun metagenomic sequencing. No significant difference was observed in the alpha diversity (represented by the Shannon diversity index) between the two groups at G100d and L23d, but the alpha diversity of sows of the probiotic group was significantly higher than that of the control group at G113d (*P* < 0.05; Wilcoxon test; Fig. 2a). Similarly, significant differences in the beta diversity (analyzed by PCoA and Adonis) of the two groups of sows were only found at G113d (R^2^ = 0.044, *P* = 0.001; Fig. 2b) but not at G100d (R^2^ = 0.036, *P* = 0.06; Fig. 2b) or L23d (R^2^ = 0.014, *P* = 0.4; Fig. 2b), indicating a more obvious effect of probiotics on modulating the sow gut microbiome at late gestation, especially near delivery period.

Then, the species-level fecal microbial microbiota composition of sows was analyzed. A total of 339 species-level genome bins (SGBs) were identified across all samples, and 16 of them were major SGBs of relative abundance >0.5% (Supplementary Table 4). To pinpoint specific changes in the gut bacteria of sows in late gestation and lactation at a finer level, responsive SGBs were identified and tracked. Responsive SGBs were defined as those displaying no significant difference in abundance between the two groups at G100d but became differentially abundant at G113d and L23d. Interestingly, sixty-two and three differential SGBs between the probiotic and control groups were identified at G113d and L23d, respectively. At 113 days of gestation, the probiotic group had significantly more *Parabacteroides distasonis*, *Prevotella tannerae*, *Alloprevotella* sp., *Porphyromonadaceae* bacterium, and *Rikenellaceae* bacterium_1 compared with the control group, while an opposite trend was observed in the species, *Clostridium sartagoforme*, *Oscillibacter* sp., and *Treponema* sp. (*P* < 0.05 in all cases; Fig. 2c). At 23 days of lactation, significantly more *Butyricicoccus porcorum* was detected in the probiotic group compared with the control group, while the abundances of *Ruminococcaceae* bacterium_4 and *Campylobacter lanienae* showed an opposite trend (*P* < 0.05 in all cases; Fig. 2c). These results together indicated that probiotic supplementation during late gestation and lactation significantly changed the gut bacteria composition in sows.

### Probiotic supplementation modulated the predicted gut bioactive compounds and carbohydrate-active enzymes (CAZymes) of sows in late gestation and lactation

**Fig. 3:**
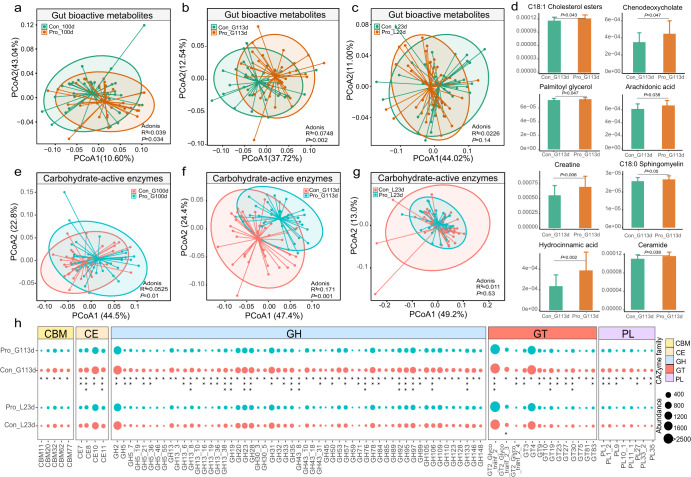
Changes in the predicted gut bioactive metabolome and carbohydrate-active enzyme (CAZyme) profile in probiotic (Pro) and control (Con) groups during the trial. Principal coordinates analysis (PCoA) score plots showing changes in the predicted gut bioactive metabolome in the two groups of sows at (**a**) 100 days of gestation (G100d), (**b**) 113 days of gestation (G113d), and (**c**) 23 days of lactation (L23d). **d** Bar charts comparing abundances of predicted differential bioactive metabolites that were responsive to the probiotic treatment. PCoA score plots showing changes in the predicted CAZyme profile in the two groups of sows at (**e**) G100d, (**f**) G113d, and (**g**) L23d. **h** Bubble plots of significant differential CAZyme subfamilies that showed a higher cumulative amount of gene in the probiotic group at G113d compared with the control group. Data were presented as means ± SEM. Subsequent changes in the gene abundance at L23d are also shown for comparison. CBM carbohydrate-binding modules, CE carbohydrate esterases, GH glycoside hydrolases, GT glycosyltransferases, and PL polysaccharide lyases. Wilcoxon test was used to evaluate statistical differences. *P* values were corrected by the Benjamini-Hochberg procedure *P < 0.05, **P < 0.01, ***P < 0.001. The center line of the box plot represents the median, the bounds of box represent IQR, and whiskers represent data other than the upper and lower quartiles.

The gut bioactive compounds of sows’ fecal samples were predicted using the MelonnPan pipeline, and a total of 80 metabolites were identified. Significant differences were observed in the gut bioactive compound profile between the probiotic and control groups during late gestation, especially soon before delivery, i.e., G100d (R^2^ = 0.039, *P* = 0.034; Fig. 3a) and G113d (R^2^ = 0.075, *P* = 0.002; Fig. 3b), but not at 23 days of lactation, i.e., L23d (R^2^ = 0.023, *P* = 0.14; Fig. 3c). The larger difference in the predicted gut active metabolite between the two groups only at the later time point of gestation was supported by the results of abundance analysis of individually predicted metabolites. A total of 16 differentially abundant metabolites between the probiotic and control groups were predicted, which showed significant intergroup differences at least at one time point (G113d and/or L23d), but not at G100d. The majority (15) of the differential abundant metabolites were identified at 113 days of gestation, while the remaining ones were identified at 23 days of lactation (Supplementary Table 5). Several probiotic-responsive metabolites, including chenodeoxycholate, arachidonic acid, C18:0 sphingomyelin, and ceramide, showed a significantly higher average predicted abundance in the probiotic group compared with the control group (*P* < 0.05; Fig. 3d). Then, we explored the probiotic-specific effect on gut metabolic modules (GMMs) by mining the metagenomic potential of sow gut microbiota in degrading and metabolizing common polysaccharides, returning 26 polysaccharide metabolism- and SCFA biosynthesis-related modules. We further calculated and comparatively analyzed the cumulative abundance of GMMs across the 339 SGBs of the two groups, revealing significantly more genes encoding modules of pectin degradation II, trehalose degradation, starch degradation, isovaleric acid synthesis II, and acetate synthesis in the probiotic group compared with the control group at G113d (*P* < 0.05; Wilcoxon test; Supplementary Fig. 2a).

To explore the enzyme repertoire for complex polysaccharide metabolism, CAZyme-encoding genes present in the sow fecal microbiome were identified. A total of 22,884 CAZyme-encoding genes were found across 339 SGBs (Supplementary Table. 6), and most genes encoded the family glycoside hydrolases (GHs, 12,727 genes), followed by glycosyltransferases (GTs, 5845 genes) and carbohydrate esterases (CEs, 2707 genes). The diversity of CAZyme in the probiotic and control groups was compared based on the cumulative abundance of CAZymes calculated from their distribution across SGBs. Interestingly, PCoA analysis of the CAZyme profile revealed a similar trend of change as the predicted gut bioactive metabolites, characterized by significant intergroup differences only during late gestation, G100d (R^2^ = 0.053, *P* = 0.01; Fig. 3e) and G113d (R^2^ = 0.171, *P* = 0.001; Fig. 3f), but not at 23 days of lactation, L23d (R^2^ = 0.011, *P* = 0.53; Fig. 3g). Comparative analysis of the abundance of individual subfamilies of the two groups revealed 116 probiotic-responsive CAZyme-encoding subfamilies at G113d and L23d, while their abundances were no significant difference between the two groups at G100d (Supplementary Table 7). Moreover, at G113d, the cumulative abundance of genes encoding 78 CAZyme subfamilies was significantly higher in the probiotic group compared with the control group; and most of them belonged to the families of GHs, PLs, and CEs (Fig. 3h). We then predicted the substrates corresponding to these enzyme families, identifying seven common substrates. At G113d, the cumulative abundance of CAZyme-encoding genes involved in the metabolism of inulin, pectin, and starch was significantly higher in the probiotic group than in the control group (*P* < 0.05; Wilcoxon test; Supplementary Fig. 2b), indicating that the probiotic intervention likely promoted pectin and starch degradation in the intestine of the sows.

### Probiotic administration modulated the gut phageome profile of sows in late gestation and lactation

**Fig. 4:**
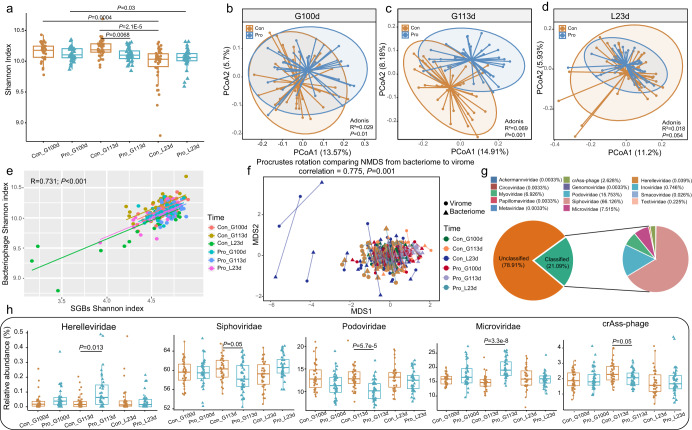
Changes in the gut phageome in probiotic (Pro) and control (Con) sows from late gestation to lactation. **a** Changes in Shannon diversity index at 100 and 113 days of gestation (G100d, G113d) and 23 days of lactation (L23d). **c**, **d** Principal coordinates analysis (PCoA) of the gut phageome of the two groups at the three time points. Results of Adonis tests are shown in the right lower corner of the plots. **e** Correlation between the values of the Shannon diversity index of the gut phageome and species-level genome bins (SGBs) profile. A strong positive correlation was found (R = 0.731; *P* < 0.001). **f** Procrustes analysis was performed on the SGBs profile and phageome of the two groups at three time points (i.e., G100d, G113d, and L23d), confirming positive cooperativity between the gut bacterial microbiota and bacteriophages (correlation = 0.775; *P* = 0.001). **g** Family-level taxonomic distribution of the phageome metagenome. **h** Wilcoxon test was used to evaluate significant differential bacteriophages between the two groups at different time points. *P* values were corrected by the Benjamini-Hochberg procedure, and a *P* value below 0.05 was considered statistically significant. Data were expressed as the means ± SEM. The center line of the box plot represents the median, the bounds of box represent IQR, and whiskers represent data other than the upper and lower quartiles.

The fecal phageome of the sows was profiled using two advanced bioinformatics tools (VIBRANT and CheckV), and 145,589 nonredundant viral OTUs (vOTUs) were detected. No significant difference was observed in the alpha diversity between the two groups at G100d and L23d, but the Shannon index of sow fecal bacteriophage of the probiotic group was significantly lower than that of the control group at G113d (*P* < 0.001; Wilcoxon test; Fig. 4a). Similar to the trends of changes in the gut bioactive metabolites and CAZymes profiles, the fecal phageome exhibited significant intergroup differences at both G100d (R^2^ = 0.029, *P* = 0.01; Fig. 4b) and G113d (R^2^ = 0.059, *P* = 0.001; Fig. 4c), but the difference was more pronounced at the later time point during gestation. However, the intergroup difference narrowed down at 23 days of lactation, L23d (R^2^ = 0.018, *P* = 0.054; Fig. 4d). Notably, the alpha diversity of the gut bacteria correlated strongly with that of the gut bacteriophage (R = 0.731, *P* < 0.001; Fig. 4e). Consistently, the Procrustes analysis also found a strong correlation between the beta diversity of the gut bacteria community and gut phageome community (correlation = 0.775; *P* = 0.001; Fig. 4f). These results suggested a high infectious specificity of gut bacteriophage toward their bacterial hosts.

Our fecal phageome dataset was then annotated by comparing it against the Metagenomic Gut Virus catalog, assigning 30,711 vOTUs (i.e., 21.09% of the 145,589 vOTUs in the current dataset) into 14 known bacteriophage families. The most dominant bacteriophage families in the sow fecal phageome were *Siphoviridae* (66.126%), *Podoviridae* (15.753%), and *Microviridae* (7.515%; Fig. 4g). Further analysis of the known bacteriophages identified five probiotic-responsive bacteriophage families that were differentially abundant between the probiotic and control groups. At 113 days of gestation, significantly more *Herelleviridae* and *Microviridae* were detected in the probiotic group than in the control group, while *Siphoviridae*, *Podoviridae*, and crAss-phage showed an opposite trend (*P* < 0.05; Fig. 4h). These results suggested that probiotic supplementation in late gestation could significantly change the gut bacteriophage composition in sows.

### Probiotic administration modulated serum metabolites of sows in late gestation and lactation

**Fig. 5:**
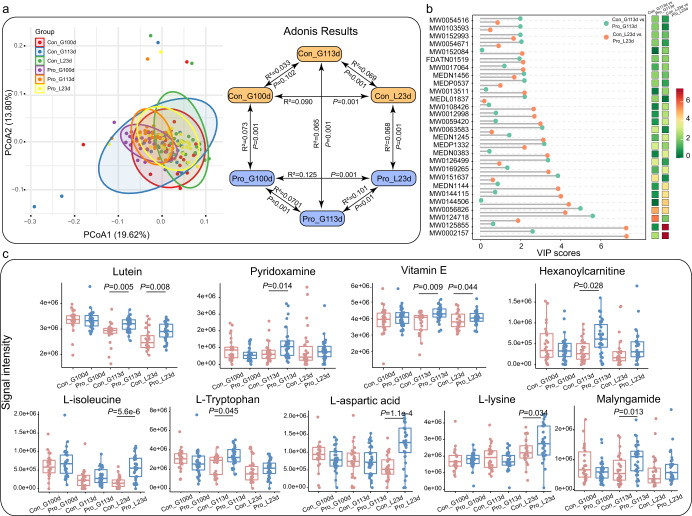
Comparison between serum metabolomes of the probiotic (Pro) and control (Con) groups at different time points. **a** Principal coordinates analysis (PCoA) of serum metabolomes of the two groups at 100 and 113 days of gestation (G100d and G113d) and 23 days of lactation (L23d). Statistical differences in the sow serum metabolome between the probiotic and control groups at different time points were evaluated by Adonis tests. **b** Significant differential metabolites were identified between the two groups at different time points. Significant differential metabolites were only identified at G113d and L23d but not G100d (cut-off: variable importance in projection [VIP] score > 2; *P* < 0.05). **c** Boxplots comparing the serum levels of probiotic-responsive metabolites. Wilcoxon test *P* values were corrected with the Benjamini-Hochberg procedure, and corrected *P* < 0.05 was considered statistically significant. Data were expressed as the means ± SEM. The center line of the box plot represents the median, the bounds of box represent IQR, and whiskers represent data other than the upper and lower quartiles.

To further reveal the probiotic-specific physiological response of sows, intergroup differences in the serum metabolome were compared at G100d, G113d, and L23d. The serum metabolomic data were analyzed with PCA, and symbols representing QC samples were tightly clustered on the PCA plot, indicating that the LC-MS system and conditions were robust, generating reliable data for downstream analysis (Supplementary Fig. 3). Symbols representing the serum metabolomes of the probiotic and control groups showed clear group-based clustering trends at the three monitored time points, G100d, G113d, and L23d. The serum metabolome profile of the probiotic group (R^2^ = 0.0701, *P* = 0.001; Fig. 5a) but not the control group (R^2^ = 0.033, *P* = 0.102; Fig. 5a) changed significantly from 100 days to 113 days of gestation, suggesting that there were significant probiotics-driven changes in the sow serum metabolite profile soon before delivery. To identify the exact changes in the serum metabolome during/after the probiotic intervention, an intergroup comparison of the abundance of individual metabolites was performed with a partial least-squares-discriminant analysis (PLS-DA) model (cut-off VIP > 2; Fig. 5b), identifying 28 differential abundant metabolites between probiotic and control at G113d and L23d. Twenty-two features were annotated to the level of metabolites by searching across the Blood Exposome Database. Several metabolites (e.g., pyridoxamine, tryptophan, vitamin E, aspartic acid, and lysine) were enriched in the probiotic group compared with the control group at G113d and L23d (*P* < 0.05; Wilcoxon test; Fig. 5c). These results of serum metabolomic analysis supported that the probiotic treatment exerted specific physiological effects on the sow, particularly near the expected delivery time.

### Probiotic administration modulated fecal short-chain fatty acids (SCFAs) of sows in late gestation and lactation

Carbohydrate-active enzymes are responsible for degrading complex fiber to form metabolites like SCFAs. Therefore, to validate the observed beneficial effects of the enrichment in genes in the CAZy families and SCFA pathways, gas chromatography-mass spectrometry (GC-MS) was used to determine the fecal SCFA content in late gestating and lactating sows. We analyzed the SCFA profile (comprising six SCFAs, namely acetic, propionic, isobutyric, butyric, isovaleric, and valeric acids) of 108 fecal samples (18 sows randomly selected per group, three time points). We found that the fecal levels of the monitored SCFAs were not significantly different between the probiotic and control groups at G100d. However, significantly more butyric and acetic acids were detected in the probiotic group compared with the control group at G113d (*P* < 0.05; Wilcoxon test), and significantly more acetic acid was also detected in the probiotic group than the control group at L23d (*P* < 0.05; Wilcoxon test; Supplementary Fig. 2c). These results demonstrated that probiotic administration could increase the fecal levels of some SCFAs in gestating and lactating sows.

### Correlation network analysis revealed probiotic-responsive features associated with constipation and host immunity

**Fig. 6:**
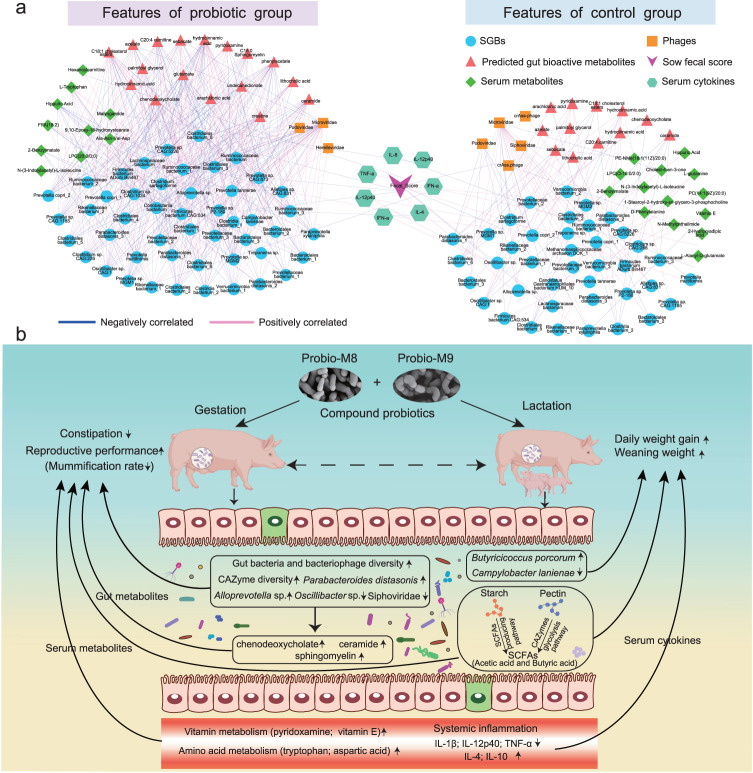
Correlation networks of monitored parameters and beneficial mechanisms of probiotic intervention for sows and piglets. **a** Integrated correlation network analysis of fecal species-level genome bins (SGBs), predicted gut bioactive metabolites, serum metabolome, sow fecal score, and serum cytokines. Correlations between datasets were evaluated by the Spearman coefficient, and features with *r* > 0.4 or *r* < −0.4 and *P* < 0.05 were selected for constructing the network plots. Blue lines represent negative correlations, and red lines represent positive correlations. **b** A proposed model of probiotic-driven beneficial changes in gut microbial species and blood metabolites responsible for alleviating constipation, lowering inflammation levels, and improving piglet growth performance. CAZyme means carbohydrate-active enzyme.

A correlation network analysis was performed to explore the association between probiotic-responsive features in the fecal bacteriome and phageome, predicted bioactive metabolome, and serum metabolome datasets in relation to constipation and immunity in sows. Our results showed that the sow fecal score correlated negatively with *Ruminococcaceae* bacterium, *Clostridia* bacterium_1, *Firmicutes* bacterium ADurb.Bin467, *Lachnospiraceae* bacterium, and *Siphoviridae* (*r* < −0.41 in all cases), and the abundance of these taxa was significantly lower in the probiotic group compared with the control group at G113d. In contrast, the abundance of several bacteria increased significantly in the probiotic group, such as *Prevotellaceae* bacterium, *Alloprevotella* sp., and *Bacteroidales* bacterium, and these taxa showed a significant positive correlation with the fecal score (*r* > 0.40 in all cases). We found that TNF-α showed a significant positive correlation with *Ruminococcaceae* bacterium_1 and *Oscillibacter* sp. (*r* > 0.40 in both cases), but a significant negative correlation with *Rikenellaceae* bacterium_1 (*r* = −0.42). The serum IL-4 level showed a significant negative correlation with *Campylobacter lanienae* (*r* = −0.43) while correlated positively with *Prevotella tannerae* (*r* = 0.41). Finally, IL-12p40 showed a significant negative correlation with two serum metabolites, i.e., malyngamide and hexanoylcarnitine (*r* < −0.40; Fig. 6a). These results suggested that probiotic treatment during late gestation and lactation could alleviate constipation and inflammatory responses of the sows by modulating their gut bacteriome and phageome and serum metabolites.

## Discussion

The health status of pregnant sows directly affects overall pig productivity. Constipation is a common problem in sows during late gestation, and constipation-induced gut dysbiosis further exacerbates oxidative stress and inflammation during parturition^1,30^. Probiotic supplementation has been shown to improve constipation and alleviate inflammation in pregnant sows, but there are few systematic longitudinal studies analyzing the severity of constipation and immune response in sows at late pregnancy and lactation in relation to piglet growth performance^31–33^. Therefore, this study investigated the beneficial effects of supplementing compound probiotics in drinking water on the health status of sows from 100 days of gestation to 23 days of lactation. Our results strongly supported that the applied probiotic formulation effectively alleviated constipation, modulated the immunity in late gestating and lactating sows, and improved piglet performance; these desirable changes were associated with the modulation of the sow serum metabolome, complex polysaccharide metabolism, gut bacteriome, and phageome (Fig. 6b).

We found that supplementing the compound probiotics in drinking water significantly relieved constipation in sows in late pregnancy and lactation, which is consistent with previous studies^33,34^. In addition, previous studies found that the serum levels of pro-inflammatory factors, such as TNF-α, IL-1β, and IL-6, in late pregnant sows increased significantly, which were closely related to constipation, abortion, fetal growth retardation, and other diseases^4,35^. Thus, we assessed the systemic inflammatory responses in sows intervened with probiotics, and observed a significant reduction in pro-inflammation markers, such as IL-1β, IL-12p40, and TNF-α, but significant increases in anti-inflammatory factors, such as IL-4 and IL-10, suggesting that the compound probiotics reduced systemic inflammation in sows during late pregnancy and lactation. Interestingly, Spearman’s correlation analysis showed that the daily gain and weaning weight of piglets had a significant positive correlation with the serum IFN-α level of sows while exhibiting a significant negative correlation with IL-8. In contrast, the serum IL-4 level showed a significant positive correlation with piglet daily gain. These findings indicated that supplementing probiotics in drinking water improved sow constipation and piglet growth performance, which may be associated with reduced systemic inflammatory responses. Recent studies have shown that the gut microbiome has a strong connection with constipation and inflammation^36,37^. The gastrointestinal microbiota plays an important role in gut motility. For example, germ-free mice have increased gastric emptying and gut transit time compared with wild-type mice^38^. Our data suggested that probiotic administration could maintain intestinal homeostasis by modulating gut microbes to reduce inflammation, thereby enhancing epithelial barrier integrity, and possibly regulating gastrointestinal motility-related endocrine responses to relieve constipation^39,40^.

Generally, a high gut microbiota diversity is considered to be desirable for maintaining a healthy physiological state^41,42^. It is thought that supplementing probiotics to sows is health-promoting, partly via building a robust gut microbiome^8^. Our study revealed that the gut bacterial microbiota of the probiotic group had a significantly higher alpha diversity compared with the control group at 113 days of gestation, and an opposite trend was observed in the diversity of gut phages. The diversity measures are the outcome of ecological processes but not the ecological process itself, and Shade et al.^43^. proposed that diversity is only “a starting point for further exploration of ecological mechanisms, rather than an ‘answer’ to community outcomes”. Therefore, although contrasting trends were observed in the alpha diversity of gut bacteria and phages, the biological meaning of such a phenomenon remains to be clarified. To explore the role of the gut bacteriome and bacteriophage, we further analyzed the structure changes of gut bacteriome and bacteriophage.

Both the gut bacteriome and phageome structure of sows however have surprisingly shown significant intra-group differences, suggesting a relatively strong effect of the probiotic intervention in shifting the overall gut microbial community structure of sows. Wang et al.^34^. supplemented *Bacillus subtilis* and *Enterococcus faecium* to sows and effectively reshaped their gut microbiota structure, which is consistent with the current results. Although the microbiota-modulating effect was more obvious on the bacteriome and less to the phageome community, one limitation to note is the lack of annotation of most bacteriophage elements in the current dataset due to insufficient knowledge and taxonomic annotation tools for gut viruses, especially in non-human subjects^44^. Nevertheless, our observation of the overall intergroup differences in the phageome structure supported that it is also part of the probiotic responsive elements and should be considered when evaluating the action of probiotic application^45^.

We further tracked significant changes in the abundance of major SGBs and bacteriophages identified in our dataset to reveal probiotic-responsive gut taxa. Our analysis revealed that the probiotic group had significantly more *Parabacteroides distasonis*, *Prevotella tannerae*, *Alloprevotella* sp., *Paraprevotella xylaniphila*, and *Rikenellaceae* bacterium, while significantly fewer *Clostridium sartagoforme*, *Oscillibacter* sp., *Treponema* sp., and *Siphoviridae*. Some of these taxa are associated with host health. For example, oral administration of *Parabacteroides distasonis* could attenuate experimental colitis in mice by modulating immunity and microbiota composition^46^. *Alloprevotella* has been considered to be beneficial bacteria as they produce succinate and acetate, which could improve the intestinal barrier and exhibit anti-inflammatory function^47^. We also found a significant positive correlation between *Alloprevotella* sp. and the sow fecal score as well as the viral family of *Microviridae*, but a significantly negative correlation was identified between *Alloprevotella* sp. and *Podoviridae*. Another taxon that correlated positively with the sow fecal score was *Paraprevotella xylaniphila*. This species has been reported to produce succinate by fermentation^48^, and its assembled genome contained multiple xylan- and pectin-hydrolyzing enzymes, which is consistent with the report of Sabater et al.^49^. identifying similar enzymes in the genome of the strain *Paraprevotella xylaniphila* YIT 11841. This strain might be associated with the alleviation of Crohn’s disease by establishing gut microbial metabolic interaction. *Siphoviridae* is a family of double-stranded DNA viruses in the order Caudovirales. In this study, a significant negative correlation was found between *Siphoviridae* and the sow fecal score, and Mihindukulasuriya et al.^50^. reported that subjects with constipation-predominant irritable bowel syndrome (IBS-C) had significantly more *Siphoviridae* than healthy subjects, suggesting a high gut *Siphoviridae* content might be linked to constipation. In addition, several responsive SGBs detected in the sow fecal microbiota were found to be significantly associated with some serum immune factors. *Oscillibacter* sp. had a significant positive correlation with the pro-inflammatory factors, IL-12p40 and IFN-α, and its abundance was significantly lower in the probiotic group compared with the control group at 113 days of gestation. Recent studies have reported that the alleviation of constipation was associated with increased abundances of beneficial bacteria, such as *Bifidobacterium* and *Alistipes*, and decreased levels of *Oscillopira* and *Odoribacter*, which are related to methanogenesis and colonic transit^51^. The results of our comparative analysis suggested that the relief of constipation and anti-inflammatory effects of compound probiotic treatment during late gestation were associated with desirable changes in the sow gut bacteriome and phageome. To quantify Probio-M8 and Probio-M9 in both control and probiotic groups, reads highly similar (>97% homology) to these strains were counted. However, we failed to detect any Probio-M8 and Probio-M9 genome across all samples using 40% breadth genome coverage, which could be due to the relatively shallow sequencing depth and thus detection power^52^. In general, at least 5-fold sequencing coverage is required for tracking a specific bacterial strain in a fecal metagenomic sample. It is interesting that although the probiotics did not engrain themselves in the microbiome at levels that were detectable via the read depth of the sequencing efforts used, it does not mean that they were not present. Particularly, it still caused detectable perturbations in other species. Comparable changes are also seen in similar gut metagenomic studies^53^.

Probiotics not only modulate host gut microbiota but also cause changes in health-related microbial metabolites^54,55^. The predicted gut bioactive metabolite profiles of the probiotic and control groups showed obvious differences at 113 days of gestation, and the abundance of several bioactive metabolites, including chenodeoxycholate, arachidonic acid, C18:0 sphingomyelin, and ceramide, increased significantly in the probiotic group. Chenodeoxycholic acid is one of the main bile acids, and two previous studies found that administering 500 to 1000 mg chenodeoxycholate in delayed-release capsules significantly accelerated colonic transit and increased stool frequency in both healthy volunteers and female patients with IBS-C^56,57^. In our study, chenodeoxycholate had a significant negative correlation with *Odoribacter*, which is known to affect colonic transit. Another potentially interesting treatment-responsive metabolite is arachidonic acid, an n-6 polyunsaturated 20-carbon fatty acid, which is beneficial to the central nervous system and the growth performance of piglets^58^. Constipation is usually accompanied by mild inflammation and damage to the intestinal barrier, and previous studies reported an important role of prostaglandins in restoring intestinal barrier function in ischemic-injured porcine ileum by converting arachidonic acid to PGH2^59,60^. In addition, our study found that arachidonic acid, sphingomyelin, and ceramide had a significant negative correlation with Clostridiales bacterium while significantly and positively correlating with *Prevotella copri* and *Prevotellaceae* bacterium. Sphingomyelins and ceramides are structural components of cellular membranes, playing critical roles in cellular signaling events^61,62^. Moreover, sphingomyelin protects against LPS-induced gut inflammation, while some very long-chain ceramides have been shown to enhance the gut barrier^63^. Our knowledge concerning specific mechanisms of the wide spectrum of lipid species in developmental programming remains extremely limited; nevertheless, our findings support that the current probiotic intervention relieved inflammation, accelerated colonic transit, and effectively relieved constipation in sows at late gestation by regulating their levels of bioactive metabolites.

The genomes of mammalian gut microbiota encode a large number of CAZymes, which are necessary for the digestion of complex polysaccharides through fermentation^64^. Our study found that the cumulative abundance of 78 of the significant differential subfamilies of CAZymes was enriched in the probiotic group compared with the control group at G113d. Many of these enzymes belonged to the families of GHs, PLs, and CEs, which are key enzymes responsible for breaking down complex carbohydrates^65^. Our study also found that the probiotic group had a significantly higher cumulative abundance of CAZyme-encoding genes involved in the metabolism of pectin and starch than in the control group, which likely enhanced the capacity of gut microbes of the probiotic group to utilize dietary complex carbohydrates directly and convert them into SCFAs or indirectly through a cross-feeding mechanism^66^. Consistently, genes encoding several SCFA production pathways were enriched in the probiotic group, accompanied by significantly more fecal butyrate and acetate levels in the probiotic group than in the control group at G113d. Subsequent increases in the intestinal content of SCFAs would further stimulate the growth of anti-inflammatory bacteria, meanwhile suppressing the pro-inflammatory bacteria in the colon^27^. These changes together could help reduce physiological inflammatory responses and improve gut health in late gestation sows.

The modulation of the gut microbiota composition would naturally accompany by changes in the colonic metabolite content, and some colonic metabolites would be absorbed and transported into and through the circulatory system to exert systemic host physiological effects^67^. Our study found interesting changes in the serum metabolome of sows receiving the probiotic treatment, i.e., enrichment in metabolites relating to vitamin metabolism (pyridoxamine, lutein, and vitamin E) and amino acid metabolism (lysine, isoleucine, tryptophan). Low levels of pyridoxamine are associated with inflammation, which is also thought to be a cause of IBS^68^. Lutein and vitamin E are powerful antioxidants that could reduce intestinal oxidation and prevent mucosal damage^69,70^. Our study observed a continuous decrease in serum lutein levels in sows from late gestation to lactation, but such a drop was slowed down with probiotic intervention, and the serum lutein levels in the probiotic recipients were significantly higher than the control sows. Moreover, recent studies found that consuming higher levels of lutein may lower the incidence of constipation^71^, and lutein has also been found to have significant anti-inflammatory effects in animal models^72^. Patients with IBS have been found to have a significantly lower serum lutein level than healthy participants, further supporting a gut health-promoting role of lutein^73^. Vitamin E is another anti-inflammatory compound that relieves constipation^74^. Our previous study found that the intake of probiotic-containing fermented milk alleviated constipation symptoms by regulating the gut microbiota, host inflammation status, and vitamin E metabolic pathways in patients, which is in line with current findings^75^. Apart from specific vitamin-associated metabolites, the serum metabolome of sows receiving the probiotic treatment had higher levels of lysine and isoleucine, tryptophan compared with those in the control group, suggesting increases in the metabolism of these amino acids. Several amino acids, including lysine, threonine, and isoleucine, have shown potential therapeutic effects on gut-related diseases and have a strong relationship with laxative effects in constipation patients^76,77^. On the other hand, tryptophan and its metabolites are mainly involved in regulating peristalsis; and probiotics play an important role in converting tryptophan to tryptamine, one of its major metabolites, that accelerates metabolism^78^. Our study found that sows in the control group exhibited a persistent decrease in serum tryptophan levels during late gestation and suffered from more severe constipation, while those in the probiotic group showed an opposite trend of change in serum tryptophan levels and did not suffer from severe constipation. Furthermore, a significant negative correlation was identified between tryptophan and *Oscillibacter* sp., and members of this genus have been shown to affect colonic transit. Thus, it is likely that the elevated levels of tryptophan helped accelerate intestinal transit in late pregnant sows. The serum tryptophan levels in patients with IBS were significantly lower than in healthy individuals, suggesting a potential role of tryptophan in maintaining colonic health^79^. Overall, the vitamin and amino acid metabolism of late gestation sows showed obvious and interesting responses after probiotic treatment, which is a key finding of the current study. We found that the addition of complex probiotics to drinking water during late gestation and lactation influenced the reproductive/growth performance of sows and piglets. In particular, the probiotic treatment significantly improved sow constipation and relieved systemic inflammation. The probiotic effects were likely achieved through orchestrated responses of the gut bacteriome, phageome, and bioactive metabolites, as well as colonic and systemic metabolism in the late gestation sows. This study provides scientific knowledge and practical information for both the academic community and the pig farming industry, expanding the scope of probiotic application in animal husbandry.

## Methods

### Experimental design

Based on similar expected dates of confinement and backfat thickness (Supplementary Table 3), 76 sows (Large × Landrace; at 100 days of gestation) were randomly allocated to the control (*n* = 38) and probiotic (*n* = 38) groups. However, two sows (sow number 7 and 13) in the probiotic group were not included in the final analysis because of the physical damage to the hind legs of sow number 7 during transfer and the unexpectedly longer farrowing period of sow number 13. All experimental diets met or exceeded the nutrient requirements of gestation and lactating sows recommended by the National Research Council (Supplementary Table 8)^80^. All sows were fed twice a day during gestation, and the amount per feeding was evaluated by a professional nutritionist on the farm, and the sows had free access to food after delivery.

The sows in the control and probiotic groups were reared in different pens in the same pig house. The drinking water of the two groups came from separate systems with the same management and water source, except that a compound probiotic formulation was added to the drinking water of the probiotic group from 100 days of gestation to 23 days of lactation. An individual meter has been installed in each sow pen to collect the sows’ daily water intake. The dosage level of probiotics was 40 grams per ton of water, comprising two bacterial strains, i.e., Probio-M8 and Probio-M9 (a total of 1 × 10^11^ CFU/g; 1:1). The compound probiotics were supplied by JinHua YinHe Biological Technology Co., Ltd. (Zhejiang, China), prepared under ISO9001 and HALAL standards in the form of dried powder.

### Isolation, cultivation, and freeze-dried powder production of probiotics

The Probio-M8 and Probio-M9 strains were isolated from breast milk samples of healthy women in Inner Mongolia in 2017^81^. Briefly, the milk samples were plated on an appropriate agar medium for isolating lactic acid bacteria, and colonies showing the morphology of lactic acid bacteria were picked and purified. The Probio-M8 and Probio-M9 strains were selected based on physiological and biochemical characterization, and their taxonomic identity was confirmed with 16 S rRNA sequencing. The strains were stored at −80 °C in the presence of a cryoprotectant (10% non-fat milk, 0.1% sodium glutamate, and 0.05% L-cysteine) and reactivated when required.

The strain reactivation was achieved by inoculating the frozen bacteria in MRS broth, incubated anaerobically at 37 °C for 24 h. The subculture was repeated twice, and the third-generation seed solution was inoculated in the same culture medium at a high-volume fraction of 7% for high-density fermentation. After high-density fermentation, the resulting culture broth was centrifuged to collect the cell pellet, which was then freeze-dried in a vacuum freeze dryer in the presence of a freeze-drying protectant to obtain the bacterial powder.

### Determination of viable counts of probiotics

The levels of live probiotics in the probiotic powder, pre-solution, diluted pre-solution, and samples collected from the drinking water system were enumerated by the pour plate method. Samples were serially diluted appropriately for pour plate counting. Inoculated plates were incubated anaerobically at 37 °C for 72 h before counting the number of colonies. A modified culture medium, MRSC modified agar (regular MRS medium supplemented with 0.5 g/L L-cysteine hydrochloride and 5.0 mg/100 mL lithium mupirocin), was used for cultivating Probio-M8; and MRS agar supplemented with 0.01% vancomycin (w/v, 10 mg/L) was used for cultivating Probio-M9.

### Feeding management

The field experiment was performed at the pig breeding site under the Zhengye Project of Inner Mongolia Zhengda Food Co., Ltd. At 110/111 days of gestation, sows were moved to a 2.2 × 1.8 m^2^ delivery bed with plastic slatted flooring, equipped with stainless steel adjustable troughs and nipples drinking fountains for sows. Each farrowing bed was equipped with a heating lamp to maintain a stable temperature (about 30 °C) in the piglet activity area. At parturition, the total number of litters, live-born, mummies, and weak piglets were recorded, and piglet birth weight was measured 12 h after farrowing.

### Fecal scores, constipation, and sample collection

The intestinal activity of the sows was monitored at six different time points, i.e., between 100 days of gestation (G100d, G106d, and G113d) and 23 days of lactation (L6d, L13d, and L19d). Sows’ feces were qualitatively evaluated before the daily morning cleaning session according to the scheme proposed by Oliviero et al. (i.e., 0 = absence of feces, 1 = dry and pellet-shaped feces, 2 = between dry and normal feces, 3 = normal and soft, but firm and well-formed feces, 4 = between normal and wet, still formed but not firm feces, and 5 = very wet, unformed, and liquid feces)^6^.

Blood and fecal samples of sows were collected at G100d, G113d, and L23d. Blood samples (10 mL) were collected in heparinized tubes from the vena jugulars of the sows, and serum samples were obtained after centrifugation at 3500 × *g* at 4 °C for 10 min, which were immediately stored at −80 °C for further analysis. Fresh fecal samples were collected from each pig using sterile 20 mL centrifuge tubes, and collected samples were stored at −80 °C for microbial composition analyses. All samples were collected by experienced veterinary personnel to avoid frightening or causing any physical harm to the sows. The lactation 23d is the experimental endpoint, and all animals survived in good health after the end of the experiment.

### Measurement of serum inflammatory factors

The serum concentrations of IFN-α, IFN-γ, IL-1β, IL-4, IL-6, IL-8, IL-10, TNF-α, and IL-12p40 were determined using sandwich enzyme-linked immunosorbent assay kits (Meimian Biotechnology, Jiangsu, China) and corresponding protocols suggested by the manufacturer.

### Metagenomic sequencing, binning, genome dereplication

Firstly, the DNA was extracted from the feces of the sows with the QIAamp Fast DNA Stool Mini Kit (Qiagen, Hilden, Germany), and metagenomic sequencing was performed on all samples on the Illumina HiSeq X Ten system. Libraries were constructed using NEBNext® Ultra™ DNA Library Prep Kit (New England Biolabs, Inc., Ipswich, MA, USA) for Illumina to produce DNA fragments of ~300 bp length. Then, reads from each sample were assembled into contigs using MEGAHIT^82^, and contigs greater than 2000 bp were selected for binning using MetaBAT2, VAMB, and DAS Tool with default parameters^83–85^. Finally, all bins were combined to obtain metagenome-assembled genomes (MAGs) using in-house scripts.

The MAGs were evaluated by CheckM and classified into MAGs of high-quality (completeness ≥80%, contamination ≤5%), medium-quality (completeness ≥70%, contamination ≤5%), and partial-quality (completeness ≥50%, contamination ≤5%)^86^. The high-quality genomes were clustered and selected by dRep to obtain SGBs, using the options -pa 0.95 and -sa 0.95^87^.

### SGBs annotation and identification of bioactive metabolites and CAZymes

The SGBs were annotated by Kraken2 against NCBI nonredundant Nucleotide Sequence Database (released in 2020.11). The relative abundance of each SGB was calculated by CoverM using the options “--min-read-percent-identity 0.95 --min-covered-fraction 0.4” (https://github.com/wwood/CoverM). The distribution of gut bioactive metabolic compounds was predicted based on the MelonnPan-predict pipeline according to the method described in our previous work^88^.

### Genome function analysis

We further analyzed the functional genome, focusing on carbohydrate degradation pathways, by implementing a module-based analytical framework (described by Valles-Colomer^89^) in which the MetaCyc metabolic database was used to predict SGBs that encoded GMMs of polysaccharide metabolism and SCFA biosynthesis pathways. The open reading frames of each SGB were predicted using with default parameters, and several methods were employed for functional annotation. Polysaccharide metabolism and SCFA biosynthesis pathways were identified based on key reactions in the Kyoto Encyclopedia of Genes and Genomes Orthologies database. Omixer-RPM (parameter: -c 0.66)^90^ was used to identify SGBs containing the related genes. dbCAN2^91^ was further used to detect CAZyme-encoding genes in the sow fecal microbiome.

### Taxonomic annotation and abundance analysis of phageome

After assembly by MEGAHIT, contigs were selected and potential viral features were identified by VIBRANT and CheckV^92^. The results recovered from these two tools were combined, and contigs greater than 5000 bp were further clustered into viral operational taxonomic units (vOTUs) with 95% nucleotide identity and 80% sequence coverage using CD-HIT (https://github.com/weizhongli/cdhit). To evaluate the novelty of vOTUs in the current dataset, 145,589 vOTUs were cross-compared with the Metagenomic Gut Virus catalog (comprised of 189,680 viral genomes from 11,810 publicly available human stool metagenomes)^93^. The average abundance of vOTUs was calculated using the CoverM-contig pipeline (https://github.com/wwood/CoverM) with the options “--min-read-percent-identity 0.95, --min-read-aligned-percent 0.5, --proper-pairs-only, and –exclude-supplementary”.

### Measurement of serum metabolites by liquid chromatography-mass spectrometry (LC-MS)

Serum samples were extracted according to Wu et al.^94^. Briefly, serum samples were thawed at 4 °C and mixed by vortexing for 10 s, and 50 μL of each sample was transferred into a fresh centrifuge tube. Three hundred microliters of acetonitrile methanol extraction solution (20%) was added and vortexed for 3 min, centrifuged at 12,000 r/min for 10 min at 4 °C. After centrifugation, 200 μL of the supernatant was transferred to another correspondingly numbered centrifuge tube and placed in a −20 °C refrigerator for 30 min, followed by a final centrifugation step at 12,000 r/min for 3 min at 4 °C. Then, 180 μL of the supernatant was pipetted into autosampler vials for analysis by mass spectrometry using AB Sciex TripleTOF 6600 (SCIEX, Framingham, MA, USA) in both positive and negative ion modes. The quality control (QC) sample was prepared by mixing the same amount of each sample, and the QC sample was injected five times before the actual analysis to evaluate the stability of the instrument.

Peak areas were corrected using the support vector machine regression method, and features with missing values of more than 50% were filtered. The metabolomic data were analyzed by PLS-DA to identify differential markers based on peak shape and signal-to-noise ratio. The marker features were cross-compared against the blood exposure database (https://bloodexposome.org) to determine the best annotation results.

### Detection of fecal SCFAs

The concentrations of fecal SCFAs were determined using a GC-MS system (TRACE 1300 Series GC System, Thermo Fisher Scientific Inc., Waltham, MA, USA) which was fitted with a capillary column Agilent HP-INNOWAX (30 m, 0.25 mm, 0.25 μm). Mass spectrometric detection of metabolites was performed on a Thermo Scientific™ ISQ™ 7000 GC-MS system. Single ion monitoring mode was used, with an electron energy of 70 eV. Fecal samples (0.1 g) were thawed, added in 2 ml of distilled water, and ultrasonically mixed for 20 min. Samples were extracted in 50 μL of 15% phosphoric acid with 10 μL of 75 μg/mL 4-methylvaleric acid solution and 140 μL ether, before centrifugation at 4 °C for 10 min at 12,000 × *g*. The supernatants were transferred into fresh sample vials before GC-MS analysis.

### Statistical analysis

All statistical analyses and data visualization were performed using the R software (v.4.1.0) and Adobe Illustrator. The species diversity, principal coordinate analysis (PCoA), PLS-DA, Adonis test, and Procrustes analysis were executed by using R packages (vegan, optparse, mixOmics, and ggpubr). Wilcoxon test and t-test were used to evaluate differences in various variables between groups, and *P* values were corrected using the Benjamini-Hochberg procedure. The cumulative abundances of GMMs modules and CAZymes were calculated by the dplyr R package with the formula: cumulative abundance = number of metabolic modules/ CAZymes encoded in the genomes × genome abundance. Correlation analyses (cut-off: *r* > 0.4 or < −0.4) of fecal scores, immune factors, differential bacterial species and bacteriophages, bioactive metabolites, and serum metabolites data were performed using the Spearman correlation coefficient. Third-party material in the figure legends was created with BioRender.com.

### Supplementary information

Supplemental Figure1, Supplemental Figure2, Supplemental Figure3, Supplemental table1-8

Reporting-summary

## Data Availability

Raw reads were deposited into the NCBI Sequence Read Archive database under (BioProject: PRJNA815745).
