# Author Correction: Probiotics alleviate constipation and inflammation in late gestating and lactating sows

**DOI:** 10.1038/s41522-023-00455-8

**Published:** 2023-11-20

**Authors:** Teng Ma, Weiqiang Huang, Yalin Li, Hao Jin, Lai-Yu Kwok, Zhihong Sun, Heping Zhang

**Affiliations:** 1 Inner Mongolia Key Laboratory of Dairy Biotechnology and Engineering, Inner Mongolia Agricultural University, Hohhot, Inner Mongolia China; 2 Key Laboratory of Dairy Products Processing, Ministry of Agriculture and Rural Affairs, Inner Mongolia Agricultural University, Hohhot, Inner Mongolia China; 3 Key Laboratory of Dairy Biotechnology and Engineering, Ministry of Education, Inner Mongolia Agricultural University, Hohhot, Inner Mongolia China

**Keywords:** Microbiome, Next-generation sequencing, Metagenomics, Microbial genetics, Policy and public health in microbiology

Correction to: *npj Biofilms and Microbiomes* 10.1038/s41522-023-00434-z, published online 23 September 2023

In the original version of this article, some Author Contributions were misattributed. This has now been corrected in both the HTML and PDF versions of the article.

