# Supplementary material for: Probiotics alleviate constipation and inflammation in late gestating and lactating sows
Source: NPJ Biofilms Microbiomes. 2023 Sep 23;9:70. doi: 10.1038/s41522-023-00434-z (PMC10517943; doi:10.1038/s41522-023-00434-z)
Supplement: Supplementary file 1 — Supplemental Figure1, Supplemental Figure2, Supplemental Figure3, Supplemental table1-8 [file 41522_2023_434_MOESM1_ESM.pdf]

Supplementary Figure 1. Pictures of feces samples of constipated sows, multi-omics correlation, and quality control for metabolite quantification. (a) Pictures of representative fecal samples of the two groups of sows were collected at different time points of gestations (100, 106, and 113 days) and lactation (6, 13, and 19 days). (b) Correlation networks between weaning weight, daily gain, and serum cytokines.

Supplementary Figure 2. Genome function analyses with focus on carbohydrate metabolism and short-chain fatty acid (SCFA) biosynthesis in different stages of gestation and lactation. (a) Differences in polysaccharide metabolism- and SCFA biosynthesis-encoding genes between the probiotic and control sows from gestation to lactation. The color scale represents the gene abundance; a greater number represents a higher abundance. (b) Predicted substrate levels of significantly differential carbohydrate-active enzyme (CAZyme)-encoding genes between the probiotic and control sows in different stages of gestation and lactation. (c) Differences in the fecal concentrations of short-chain fatty acids (SCFAs) of sows between the probiotic and the control groups. Data are presented as means ± SEM. Significant differences were evaluated by Wilcoxon tests, and a $P < 0.05$ was considered statistically significant. The sampling time points were 100 and 113 days of gestation (G100 and G113) and 23 days of lactation (L23). CT1= control group G100; ET1= probiotic group G100; CT2= control group G113; ET2= probiotic group G113; CT3= control group L23; ET3= probiotic group L23; Pro = probiotic group; Con = control group; GMM = gut metabolic module.

Supplementary Figure 3. Principal component analysis of serum metabolomes of late

gestating and lactating sows. Pro = probiotic group; Con = control group; QC = quality control samples.

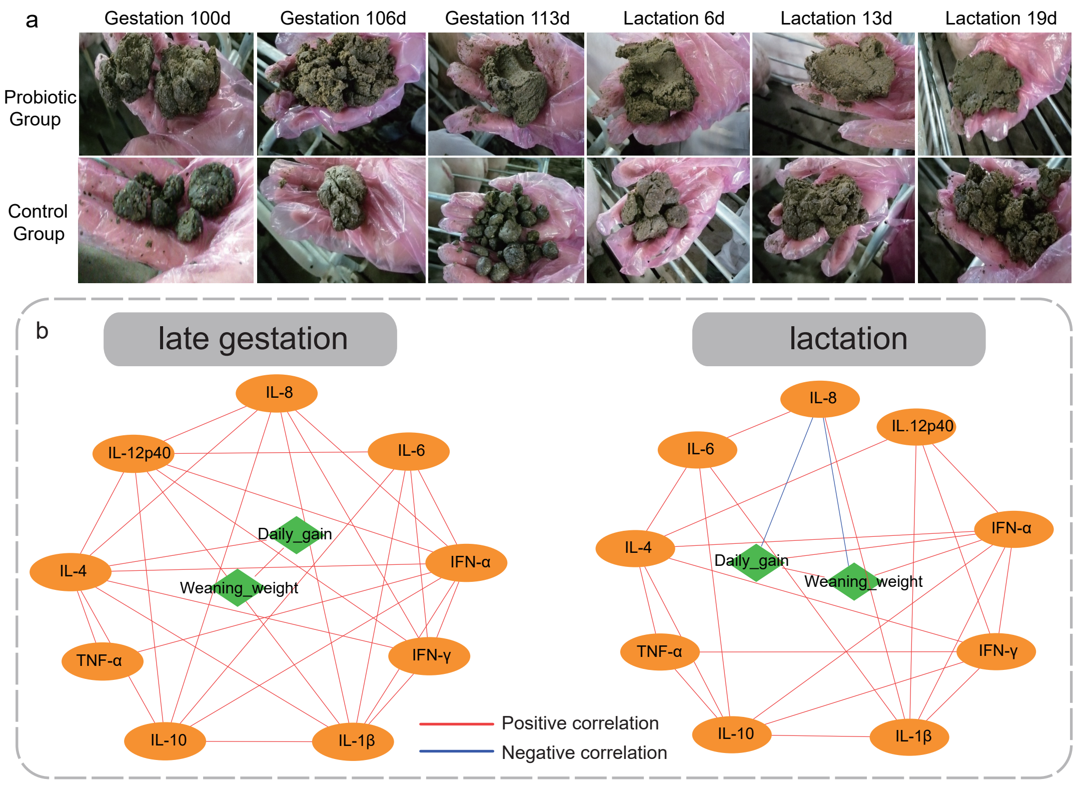
a
Gestation 100d
Gestation 106d
Gestation 113d
Lactation 6d
Lactation 13d
Lactation 19d
Probiotic Group
Control Group
b
late gestation
lactation
IL-8
IL-12p40
IL-6
Daily_gain
IL-4
IFN-α
Weaning_weight
TNF-α
IFN-γ
IL-10
IL-1β
IL.12p40
Positive correlation
Negative correlation

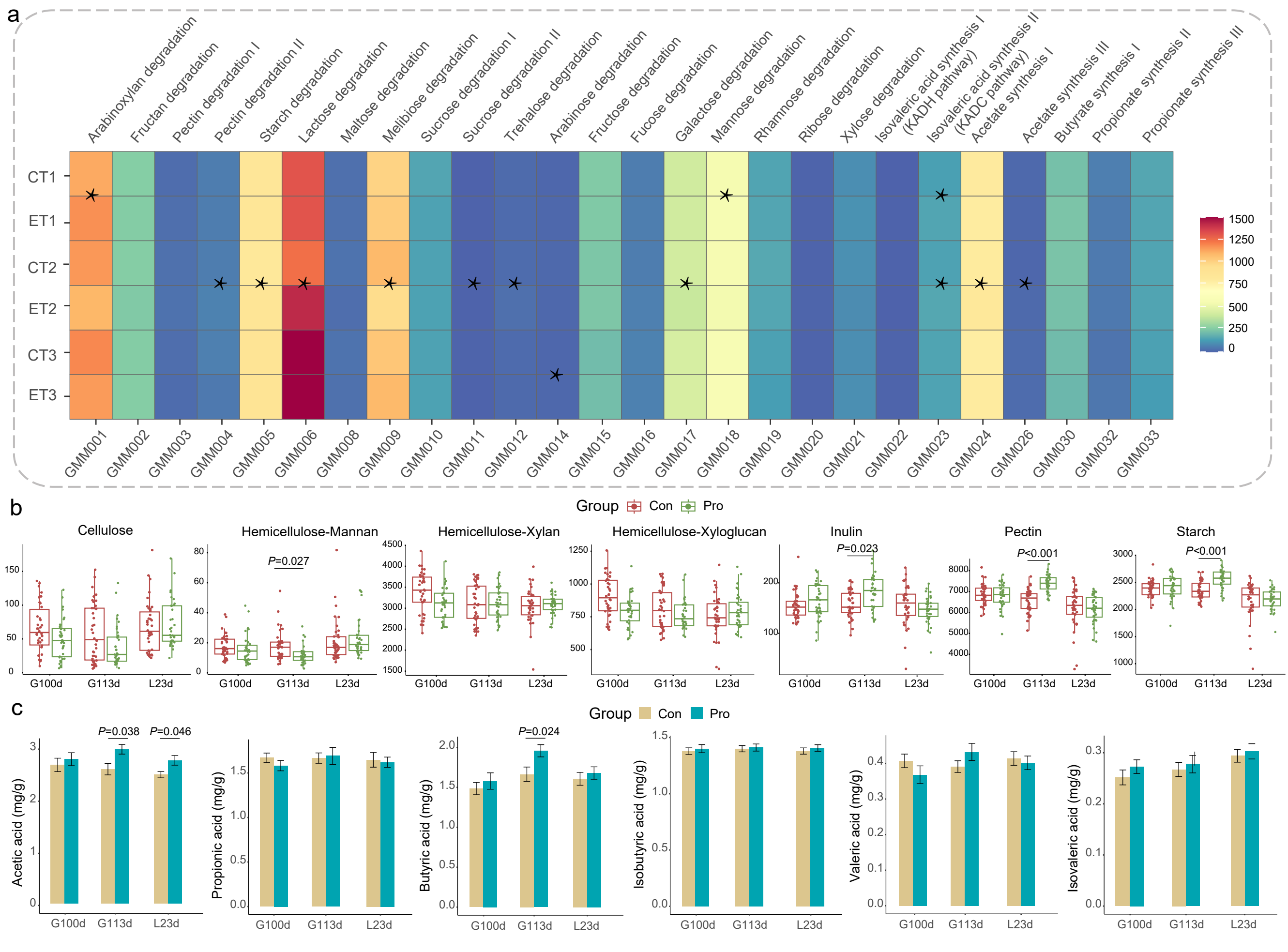
a
Arabinoxylan degradation
Fructan degradation
Pectin degradation I
Pectin degradation II
Starch degradation
Lactose degradation
Maltose degradation
Melibiose degradation
Sucrose degradation I
Sucrose degradation II
Trehalose degradation
Arabinose degradation
Fructose degradation
Fucose degradation
Galactose degradation
Mannose degradation
Rhamnose degradation
Ribose degradation
Xylose degradation
Isovaleric acid synthesis I (KADH pathway)
Isovaleric acid synthesis II (KADC pathway)
Acetate synthesis I
Acetate synthesis III
Butyrate synthesis I
Propionate synthesis II
Propionate synthesis III
CT1
ET1
CT2
ET2
CT3
ET3
GMM001
GMM002
GMM003
GMM004
GMM005
GMM006
GMM008
GMM009
GMM010
GMM011
GMM012
GMM014
GMM015
GMM016
GMM017
GMM018
GMM019
GMM020
GMM021
GMM022
GMM023
GMM024
GMM026
GMM030
GMM032
GMM033
1500
1250
1000
750
500
250
0
b
Group Con Pro
Cellulose
Hemicellulose-Mannan
Hemicellulose-Xylan
Hemicellulose-Xyloglucan
Inulin
Pectin
Starch
P=0.027
P=0.023
P<0.001
P<0.001
G100d
G113d
L23d
c
Group Con Pro
P=0.038
P=0.046
P=0.024
Acetic acid (mg/g)
Propionic acid (mg/g)
Butyric acid (mg/g)
Isobutyric acid (mg/g)
Valeric acid (mg/g)
Isovaleric acid (mg/g)
G100d
G113d
L23d

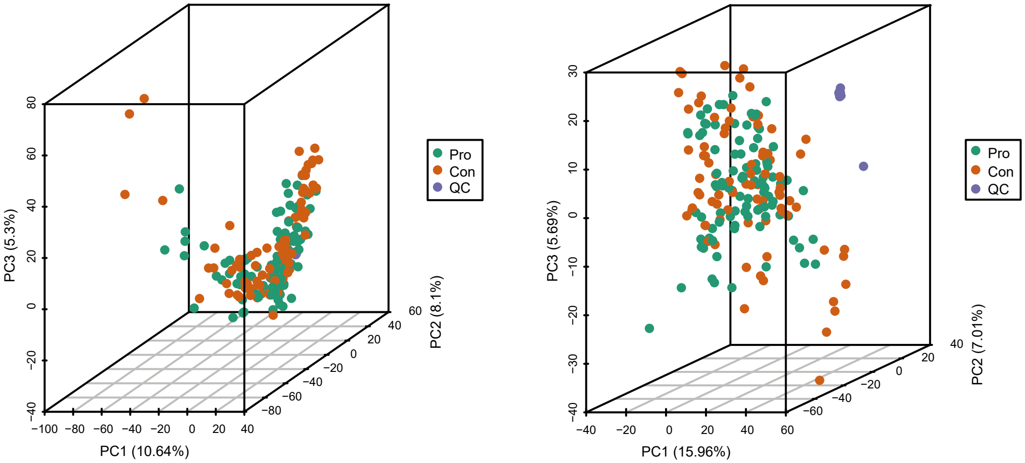

PC1 (10.64%)
PC2 (8.1%)
PC3 (5.3%)
Pro
Con
QC
PC1 (15.96%)
PC2 (7.01%)
PC3 (5.69%)
Pro
Con
QC

Supplementary table 1. Viable probiotic counts in the probiotic powder, pre-solution, diluted pre-solution, and drinking water system

Viable counts in the probiotic powder and pre-solution

	Bifidobacterium lactis Probio-M8	*Lacticaseibacillus rhamnosus* Probio-M9
Compound probiotic powder (CFU/g)	$(5.70±0.23)×10^{10}$	$(5.20±0.48)×10^{10}$
Freshly prepared 100-fold diluted pre-solution (CFU/mL)	$(6.03±0.47)×10^{8}$	$(3.44±0.35)×10^{8}$
Pre-solution standing for 1 hour (CFU/mL)	$(5.73±0.50)×10^{8}$	$(3.56±0.14)×10^{8}$
Pre-solution standing for 2 hours (CFU/mL)	$(4.55±0.43)×10^{8}$	$(3.37±0.20)×10^{8}$
Pre-solution standing for 3 hours (CFU/mL)	$(4.82±0.29)×10^{8}$	$(3.11±0.18)×10^{8}$

Viable probiotic bacterial counts in the diluted pre-solution of late gestational sows of the probiotic group

	Bifidobacterium lactis Probio-M8	*Lacticaseibacillus rhamnosus* Probio-M9
Diluted pre-solution standing for 1 hour (CFU/mL)	$(2.65±0.21)×10^{6}$	$(2.40±0.61)×10^{6}$
Diluted pre-solution standing for 2 hours (CFU/mL)	$(2.16±0.21)×10^{6}$	$(1.73±0.55)×10^{6}$
Diluted pre-solution standing for 3 hours (CFU/mL)	$(2.24±0.81)×10^{6}$	$(1.61±0.18)×10^{6}$

Viable probiotic bacterial counts in the diluted pre-solution of lactational sows of the probiotic group

	Bifidobacterium lactis Probio-M8	*Lacticaseibacillus rhamnosus* Probio-M9
Diluted pre-solution standing for 1 hour (CFU/mL)	$(3.20±0.34)×10^{6}$	$(2.00±0.27)×10^{6}$
Diluted pre-solution standing for 2 hours (CFU/mL)	$(2.84±0.18)×10^{6}$	$(2.15±0.25)×10^{6}$
Diluted pre-solution standing for 3 hours (CFU/mL)	$(3.14±0.22)×10^{6}$	$(1.69±0.14)×10^{6}$

Supplementary table 2. Comparison of average daily feed intake and water consumption between probiotic and control group from late gestation to lactation

**Average daily feed intake of sows during the trial (kg)**					
**Gestation (G) period**			**Lactation (L) period**		
Date	Probiotic group	Control group	Date	Probiotic group	Control group
G100d	2.71	2.84	L1d	4.37	5.31
G101d	2.62	2.96	L2d	7.14	5.15
G102d	3.13	3.22	L3d	4.12	5.77
G103d	2.65	2.48	L4d	5.07	5.59
G104d	2.89	2.77	L5d	5.56	4.67
G105d	2.45	3.12	L6d	9.53	6.23
G106d	2.31	2.36	L7d	5.30	5.78
G107d	2.65	2.80	L8d	5.75	4.72
G108d	3.23	2.37	L9d	5.08	5.27
G109d	2.46	2.73	L10d	5.51	5.68
G110d	2.57	2.51	L11d	5.02	6.33
G111d	2.67	2.84	L12d	5.02	5.51
G112d	2.77	2.89	L13d	4.32	6.16
G113d	2.49	2.66	L14d	5.51	4.71
G114d	2.85	2.71	L15d	3.87	6.15
**Average**	**2.70**	**2.75**	L16d	6.36	4.11
			L17d	5.19	4.37
			L18d	4.09	7.14
			L19d	5.02	4.12
			L20d	5.59	5.07
			L21d	3.81	5.56
			L22d	5.66	5.75
			L23d	5.92	5.30
			**Average**	**5.34**	**5.41**

**Average daily water intake of sows during the trial (kg)**

Gestation (G) period			Lactation (L) period		
Date	Probiotic group	Control group	Date	Probiotic group	Control group
G100d	13.63	19.48	L1d	17.86	37.04
G101d	17.22	14.94	L2d	53.57	37.04
G102d	13.63	11.78	L3d	18.52	37.74
G103d	14.07	10.49	L4d	19.61	19.23
G104d	12.63	11.06	L5d	39.22	37.04
G105d	10.32	11.49	L6d	19.61	55.56
G106d	12.12	11.69	L7d	58.82	37.04
G107d	10.37	10.81	L8d	39.22	35.71
G108d	10.79	10.30	L9d	53.57	35.71
G109d	10.94	10.40	L10d	53.57	35.71
G110d	11.92	11.54	L11d	53.57	53.57
G111d	20.97	11.89	L12d	62.50	53.57
G112d	11.00	14.31	L13d	62.50	53.57
G113d	11.19	12.83	L14d	53.57	71.43
G114d	12.09	11.63	L15d	71.43	53.57
**Average**	**12.86**	**12.31**	L16d	71.43	53.57
			L17d	53.57	53.57
			L18d	53.57	35.71
			L19d	35.71	53.57
			L20d	71.43	72.73
			L21d	54.55	36.36
			L22d	40.00	76.92
			L23d	60.00	76.92
			**Average**	**48.58**	**48.39**

Supplementary table 3. Comparison of reproductive performance of sows and growth performance of piglets between probiotic and control groups

ID	Group	Sow number	Expected date of delivery	Date of delivery	Parity	G100d Backfat thickness (mm)	Total litter size	Healthy litter rate	Stillbirth rate	Mummification rate	Weak litter rate	Treatment days of piglet diarrhea
ME01	Probiotic	182492	2019.08.24	2019.08.25	1	19.78	16	93.75%	0.00%	0.00%	6.25%	3
ME02	Probiotic	806824	2019.08.24	2019.08.26	3	19.52	13	92.31%	7.69%	0.00%	0.00%	2
ME03	Probiotic	184143	2019.08.24	2019.08.24	3	19.02	12	100.00%	0.00%	0.00%	0.00%	2
ME04	Probiotic	183189	2019.08.24	2019.08.26	1	19.76	11	90.91%	9.09%	0.00%	0.00%	2
ME05	Probiotic	806646	2019.08.24	2019.08.26	3	19.38	7	100.00%	0.00%	0.00%	0.00%	1
ME06	Probiotic	804644	2019.08.24	2019.08.25	3	19.32	12	83.33%	16.67%	0.00%	0.00%	2
ME08	Probiotic	807172	2019.08.24	2019.08.24	3	19.26	14	85.71%	14.29%	0.00%	0.00%	2
ME09	Probiotic	806208	2019.08.24	2019.08.25	3	19.21	12	100.00%	0.00%	0.00%	0.00%	2
ME10	Probiotic	804222	2019.08.24	2019.08.21	3	19.45	10	70.00%	30.00%	0.00%	0.00%	1
ME11	Probiotic	802676	2019.08.24	2019.08.25	3	19.93	16	93.75%	0.00%	0.00%	6.25%	1
ME12	Probiotic	807163	2019.08.24	2019.08.26	3	20.04	14	78.57%	0.00%	21.43%	0.00%	2
ME14	Probiotic	806751	2019.08.24	2019.08.25	3	19.77	11	90.91%	0.00%	9.09%	0.00%	2
ME15	Probiotic	804847	2019.08.24	2019.08.25	3	19.73	14	100.00%	0.00%	0.00%	0.00%	4
ME16	Probiotic	806248	2019.08.24	2019.08.25	3	19.86	14	92.86%	0.00%	0.00%	7.14%	2
ME17	Probiotic	807029	2019.08.24	2019.08.25	3	19.80	12	75.00%	0.00%	0.00%	25.00%	6
ME18	Probiotic	801673	2019.08.24	2019.08.24	3	19.23	15	100.00%	0.00%	0.00%	0.00%	2
ME19	Probiotic	184013	2019.08.24	2019.08.25	1	19.16	10	100.00%	0.00%	0.00%	0.00%	4
ME20	Probiotic	806842	2019.08.24	2019.08.25	3	19.79	15	100.00%	0.00%	0.00%	0.00%	1
ME21	Probiotic	801106	2019.08.24	2019.08.25	3	19.92	14	92.86%	7.14%	0.00%	0.00%	3
ME22	Probiotic	804579	2019.08.24	2019.08.24	3	19.15	15	80.00%	13.33%	0.00%	6.67%	4
ME23	Probiotic	184835	2019.08.24	2019.08.25	1	19.98	10	100.00%	0.00%	0.00%	0.00%	2
ME24	Probiotic	803863	2019.08.24	2019.08.24	3	20.01	16	93.75%	0.00%	6.25%	0.00%	1
ME25	Probiotic	806126	2019.08.24	2019.08.24	3	19.04	23	78.26%	0.00%	0.00%	4.35%	1
ME26	Probiotic	805740	2019.08.24	2019.08.25	3	19.47	17	76.47%	11.76%	5.88%	5.88%	3
ME27	Probiotic	804928	2019.08.24	2019.08.25	3	19.10	16	87.50%	6.25%	0.00%	0.00%	2
ME28	Probiotic	802739	2019.08.24	2019.08.23	3	19.53	14	92.86%	7.14%	0.00%	0.00%	3
ME29	Probiotic	806249	2019.08.24	2019.08.25	3	19.17	17	88.24%	11.76%	0.00%	0.00%	2
ME30	Probiotic	806684	2019.08.24	2019.08.25	3	19.56	20	95.00%	0.00%	0.00%	5.00%	3
ME31	Probiotic	806788	2019.08.24	2019.08.25	3	19.45	20	70.00%	25.00%	0.00%	5.00%	3
ME32	Probiotic	803700	2019.08.24	2019.08.25	3	19.59	17	100.00%	0.00%	0.00%	0.00%	3
ME33	Probiotic	806135	2019.08.24	2019.08.24	3	20.05	15	100.00%	0.00%	0.00%	0.00%	4

ME34	Probiotic	806220	2019.08.24	2019.08.25	3	19.34	9	77.78%	0.00%	0.00%	11.11%	1
ME35	Probiotic	804340	2019.08.24	2019.08.25	3	19.63	14	92.86%	0.00%	7.14%	0.00%	2
ME36	Probiotic	800787	2019.08.24	2019.08.25	3	19.94	16	87.50%	12.50%	0.00%	0.00%	1
ME37	Probiotic	803271	2019.08.24	2019.08.23	3	19.24	17	94.12%	0.00%	5.88%	0.00%	3
ME38	Probiotic	804499	2019.08.24	2019.08.25	3	19.54	15	100.00%	0.00%	0.00%	0.00%	2
MC01	Control	803725	2019.08.09	2019.08.10	3	19.89	18	100.00%	0.00%	0.00%	0.00%	2
MC02	Control	802597	2019.08.09	2019.08.09	3	19.05	18	83.33%	11.11%	5.56%	0.00%	2
MC03	Control	801911	2019.08.09	2019.08.09	3	19.45	14	100.00%	0.00%	0.00%	0.00%	1
MC04	Control	803416	2019.08.09	2019.08.08	3	20.01	14	100.00%	0.00%	0.00%	0.00%	2
MC05	Control	804017	2019.08.09	2019.08.09	1	19.34	15	86.67%	0.00%	13.33%	0.00%	3
MC06	Control	803921	2019.08.09	2019.08.09	3	19.15	11	90.91%	0.00%	9.09%	0.00%	4
MC07	Control	802145	2019.08.09	2019.08.09	1	19.30	15	73.33%	13.33%	6.67%	6.67%	5
MC08	Control	804779	2019.08.09	2019.08.09	3	19.84	15	86.67%	0.00%	13.33%	0.00%	2
MC09	Control	802557	2019.08.09	2019.08.09	3	19.02	10	100.00%	0.00%	0.00%	0.00%	2
MC10	Control	804637	2019.08.09	2019.08.09	3	19.67	16	93.75%	6.25%	0.00%	0.00%	2
MC11	Control	804602	2019.08.09	2019.08.09	3	19.43	10	100.00%	0.00%	0.00%	0.00%	3
MC12	Control	803533	2019.08.09	2019.08.09	3	20.03	15	80.00%	20.00%	0.00%	0.00%	2
MC13	Control	803246	2019.08.09	2019.08.09	3	19.04	18	83.33%	11.11%	0.00%	5.56%	7
MC14	Control	803807	2019.08.09	2019.08.09	3	19.39	15	86.67%	13.33%	0.00%	0.00%	2
MC15	Control	803706	2019.08.09	2019.08.09	3	19.83	16	75.00%	0.00%	25.00%	0.00%	4
MC16	Control	803868	2019.08.09	2019.08.09	3	19.36	11	100.00%	0.00%	0.00%	0.00%	4
MC17	Control	803913	2019.08.09	2019.08.09	3	19.78	16	100.00%	0.00%	0.00%	0.00%	5
MC18	Control	807167	2019.08.09	2019.08.09	3	19.59	9	100.00%	0.00%	0.00%	0.00%	1
MC19	Control	801551	2019.08.09	2019.08.09	3	19.56	14	85.71%	0.00%	7.14%	7.14%	1
MC20	Control	182549	2019.08.09	2019.08.09	1	19.13	13	92.31%	0.00%	7.69%	0.00%	2
MC21	Control	803753	2019.08.09	2019.08.09	3	19.29	11	100.00%	0.00%	0.00%	0.00%	1
MC22	Control	806027	2019.08.09	2019.08.08	3	19.82	16	81.25%	6.25%	12.50%	0.00%	4
MC23	Control	800262	2019.08.09	2019.08.09	3	19.72	13	92.31%	7.69%	0.00%	0.00%	2
MC24	Control	190199	2019.08.09	2019.08.09	1	19.52	15	93.33%	0.00%	6.67%	0.00%	0
MC25	Control	804451	2019.08.09	2019.08.09	3	19.49	10	80.00%	20.00%	0.00%	0.00%	0
MC26	Control	802402	2019.08.09	2019.08.09	3	19.32	10	80.00%	20.00%	0.00%	0.00%	0
MC27	Control	806004	2019.08.09	2019.08.09	3	19.12	15	86.67%	6.67%	0.00%	6.67%	2
MC28	Control	804434	2019.08.09	2019.08.09	3	19.19	17	94.12%	0.00%	5.88%	0.00%	3
MC29	Control	804061	2019.08.09	2019.08.09	3	19.81	16	93.75%	0.00%	6.25%	0.00%	2
MC30	Control	807013	2019.08.09	2019.08.09	3	19.71	17	82.35%	17.65%	0.00%	0.00%	1
MC31	Control	801822	2019.08.09	2019.08.09	3	19.61	12	100.00%	0.00%	0.00%	0.00%	1
MC32	Control	802102	2019.08.09	2019.08.09	3	19.42	10	100.00%	0.00%	0.00%	0.00%	1
MC33	Control	800903	2019.08.09	2019.08.09	1	19.12	13	76.92%	7.69%	7.69%	7.69%	6

MC34	Control	802618	2019.08.09	2019.08.09	3	19.11	14	92.86%	7.14%	0.00%	0.00%	1
MC35	Control	189146	2019.08.09	2019.08.09	1	19.06	12	91.67%	8.33%	0.00%	0.00%	5
MC36	Control	802349	2019.08.09	2019.08.10	3	19.90	11	100.00%	0.00%	0.00%	0.00%	3
MC37	Control	804491	2019.08.09	2019.08.09	3	19.72	20	75.00%	0.00%	25.00%	0.00%	4
MC38	Control	804278	2019.08.09	2019.08.10	3	19.05	20	95.00%	0.00%	0.00%	5.00%	4

Supplementary table 4. Species with relative abundance greater than 0.5% in the sow fecal microbiota

**Genome_ID**	SGB_47	SGB_28	SGB_92	SGB_11	SGB_89	SGB_44	SGB_2	SGB_80	SGB_6	SGB_42	SGB_65	SGB_3	SGB_200	SGB_29	SGB_32	SGB_140
Relative abundance (%)	1.39	1.02	0.99	0.96	0.91	0.86	0.83	0.78	0.75	0.75	0.74	0.68	0.63	0.59	0.56	0.55
Taxonomy	Bacteroides sp. 43_108	Bacteroidales bacterium WCE2004	Oscillibacter sp.	Marinilabiliaceae bacterium JC017	Methanobrevibacter gottschalkii	Treponema sp.	Clostridia bacterium	Lachnospiraceae bacterium	Treponema bryantii	Bacteroidales bacterium	Prevotella sp. P5-92	Rikenellaceae bacterium_4	Rikenellaceae bacterium_2	Clostridiales bacterium	Bacteroides sp.	Rikenellaceae bacterium_3
Sample_MC01_1	3.7	2.895	0.47	0.264	2.39	0.814	0.644	1.54	1.492	0.176	0.23	1.06	2.36	0.444	1.45	0.442
Sample_MC01_2	3.03	2.579	0.666	0.163	2.166	0.563	0.813	1.2	2.737	0.34	0.47	0.72	2.19	0.482	0.81	0.412
Sample_MC01_3	0.83	1.129	0.468	1.074	1.799	1.159	1.087	0.74	1.073	0.909	0.67	0.85	0.59	0.216	1.46	0.319
Sample_MC02_1	2.89	2.962	1.337	0.354	0.03	1.521	0.61	0.35	1.456	0.302	0.39	0.54	0.41	0.356	0.65	0.187
Sample_MC02_2	2.29	2.447	1.494	0.784	0.251	1.674	1.284	0.41	1.461	0.994	0.46	0.7	0.3	1.583	0.74	0.244
Sample_MC02_3	2.19	1.733	1.515	0.428	0	0.915	0.453	0.35	2.302	0	0.41	0	0.31	1.241	0.53	0.083
Sample_MC03_1	2.45	1.517	1.207	1.966	0.864	1.161	0	1.37	1.037	0.169	0.61	0.53	0.43	0.434	0.27	0.761
Sample_MC03_2	0.63	0.593	1.627	0.544	1.379	0.065	0.607	0.79	1.319	0.58	0.51	0.48	0.98	0.831	0.24	0.343
Sample_MC03_3	1.67	2.055	1.51	0.294	1.543	0.145	6.153	1.07	0.393	0	0.02	0.37	1.21	1.301	0.25	0.069
Sample_MC04_1	0	0.178	0.768	1.137	0.465	0.685	0.448	0.66	0	0.792	0.62	0.82	0.49	0.205	0.16	0.812
Sample_MC04_2	0	0.034	1.295	0.121	0.038	0.096	0	0.53	0	3.005	0.53	2.51	0	0.988	0.63	0.457
Sample_MC04_3	0.42	0.812	0.992	1.264	0.485	0.112	0.452	0.42	0	0.286	0.87	4.37	0.14	1.076	0.37	0.219
Sample_MC05_1	0	0.26	0.24	0.572	0	0.487	0	0.55	0.053	1.176	0.48	0.51	0	0.245	0.44	0.663
Sample_MC05_2	0	0.32	0.589	0.34	0	0.318	0	1.31	0.049	0.275	0.77	0.54	0.05	0.096	0.8	1.949
Sample_MC05_3	0	0.661	2.048	0.923	0	1.735	0.549	0.45	0	0.985	1.09	0.44	0	0.916	0.43	0.344
Sample_MC06_1	2.05	0.447	0.595	2.29	1.736	0.467	0.496	1.17	0.091	0.123	0.74	0.67	1.08	0.775	1.12	0.415
Sample_MC06_2	1.84	0.156	0.458	0.181	1.727	0.41	0	0.84	0.059	0.714	1.24	0.71	0.84	0.986	1.09	0.812
Sample_MC06_3	0	0	0.682	0.252	1.347	0.385	0	0.7	0.197	0.391	1.42	0.5	0.21	0.034	1.35	0.23
Sample_MC07_1	3.02	1.368	1.41	0.632	0.667	0.607	0.555	0.54	0.955	0.932	0.8	0	0.08	0.712	0.66	0.49
Sample_MC07_2	0	0.311	1.877	0.251	1.904	0.406	0	0.55	3.379	0.448	0.84	0	1.21	0.388	0.77	0.099
Sample_MC07_3	2.07	0.472	0.963	0.665	0.088	0.559	0.783	0.29	1.608	0.478	0.84	0	0.2	0.792	0.78	0.312
Sample_MC08_1	2.71	0.886	0.348	1.624	1.718	1.451	0.528	0.68	0	1.781	1.01	0.57	0.84	0.21	0.56	0.81
Sample_MC08_2	1.66	1.388	0.753	1.243	1.766	1.472	0.518	0.97	0.096	0.414	0.78	0.69	0.21	0.213	0.33	0.842
Sample_MC08_3	0.65	0.553	0.279	1.222	1.084	1.748	0.575	1	0.22	0.781	0.81	0.39	0.42	0.351	0.33	0.73

Sample_MC09_1	1.74	0.656	1.206	1.363	1.244	1.489	0.489	0.6	1.28	0.753	1.14	0.55	0.29	0.542	0.75	0.71
Sample_MC09_2	1.49	0.709	1.51	1.236	1.201	0.953	0	1.07	0.203	0.699	0.67	0.63	0.11	0.782	0.32	0.63
Sample_MC09_3	1.8	2.876	0.743	1.038	0.026	1.651	0.575	0.68	3.179	1.425	0.58	0.71	0.1	0.311	0.74	0.411
Sample_MC10_1	0.86	0.552	1.379	0.411	1.373	0.752	0.595	0.98	0.404	1.24	0.41	0.56	0.55	0.292	0.84	0.912
Sample_MC10_2	0.38	0.459	0.72	0.509	0.365	0.52	0.775	0.55	0.225	1.37	0.5	1.38	0.29	0.623	0.19	1.511
Sample_MC10_3	0.71	0.713	0.419	0.425	0.472	1.573	0.571	0.47	0.896	1.012	0.83	1.02	0.09	0.529	0.94	0.378
Sample_MC11_1	2.48	1.531	0.464	0.264	0.673	1.268	0.51	0.9	0.469	1.091	0.54	0.55	1.64	0.798	0.91	0.428
Sample_MC11_2	1.32	0.697	1.182	0.791	1.563	1.514	0.839	0.76	0.501	0.508	0.89	1.32	0.32	0.308	0.83	0.607
Sample_MC11_3	2.52	0.724	1.616	0.336	1.85	0.189	1.086	2.32	1.204	0.144	0.38	0.63	0.47	0.816	0.4	0.237
Sample_MC12_1	2.14	2.256	0.964	1.073	2.022	1.582	0.509	0.86	0.919	0.404	0.49	0.51	1.68	0.46	1.45	0.432
Sample_MC12_2	1.3	0.702	0.479	1.652	1.134	1.157	0.548	1.02	0.304	0.502	0.65	0.55	0.7	0.497	0.68	1.114
Sample_MC12_3	2.01	0.701	0.277	1.737	0.867	2	0.587	1.2	0.277	1.122	2.02	1.02	0.18	0.321	0.47	0.516
Sample_MC13_1	2.59	1.791	1.755	0.662	1.076	0.981	0.524	1.01	0.232	0.177	0.35	0.52	0.82	1.594	0.74	0.572
Sample_MC13_2	3.27	1.396	1.524	1.087	1.649	2.025	0.574	0.99	0.182	0.208	0.65	0.83	0.98	0.903	0.59	0.339
Sample_MC13_3	1.92	1.163	0.652	2.865	0.48	1.279	0.687	0.63	0.34	0.142	0.62	0.51	0.34	0.359	0.51	0.265
Sample_MC14_1	0.57	0.515	0.587	0.253	0.796	0.427	0.443	0.61	0	0.98	0.63	1.33	0.16	0.343	0.69	1.169
Sample_MC14_2	0.44	0.686	0.593	0.5	0.388	0.842	0.552	1.18	0.157	2.921	0.75	0.83	0.21	0.821	0.33	0.454
Sample_MC14_3	1.56	0.111	0.517	0.418	0.769	0.344	5.393	0.4	0.058	0.156	0.89	1.74	0.15	0.447	0.32	0.445
Sample_MC15_1	3.16	3.946	1.119	0.3	1.348	0.942	0.546	0.82	0.436	0.454	0.65	0.86	0.58	0.223	1.4	0.801
Sample_MC15_2	2.95	1.361	1.037	0.533	1.11	0.785	0.658	0.75	1.328	0.47	0.93	2.35	0.43	0.34	0.61	0.62
Sample_MC15_3	0.9	1.846	0.751	0.797	1.686	1.274	0.44	0.85	0.478	0.333	0.98	0.86	0.16	0.95	0.54	0.326
Sample_MC16_1	2.29	2.239	1.93	0.809	1.871	1.108	1.067	1.11	1.816	0.059	0.25	0.65	1.78	0.841	0.73	0.194
Sample_MC16_2	2.52	1.891	0.94	0.958	0.99	1.89	0.678	0.97	0.571	0.045	0.38	0.68	1.28	0.965	0.86	0.245
Sample_MC16_3	0.59	0.617	0.944	1.163	0.952	1.007	0.589	1.05	0.436	0.049	0.71	0.39	0.65	0.982	0.34	0.315
Sample_MC17_1	2.49	2.064	0.997	1.245	0.808	0.945	0.701	0.8	1.541	0.619	0.92	0.72	0.54	0.284	0.45	0.623
Sample_MC17_2	1.75	1.366	1.087	1.072	0.871	1.033	0.52	0.83	1.581	0.102	0.98	0.77	0.65	0.451	0.47	0.329
Sample_MC17_3	1.47	0.541	1.502	1.141	0.684	1.091	0.512	1.24	1.806	0.076	0.79	0.47	0.76	0.693	0.22	0.136
Sample_MC18_1	1	0.481	0.533	0.49	0.452	1.098	0.404	0.75	0.166	1.102	0.77	0.85	0.45	0.387	0.34	0.386
Sample_MC18_2	0.51	0.175	0.459	0.237	0.539	0.274	0.592	1.1	0	1.659	0.8	0.95	0.52	0.294	0.25	1.207
Sample_MC18_3	0.9	0.462	1.62	1.358	0.548	0.823	2.082	0.61	0.116	0.038	1.57	1.26	0.23	0.783	0.36	0.141
Sample_MC19_1	0.35	0.102	1.209	0.223	0.773	0.905	0.46	0.72	0	1.952	1.28	0.62	0.09	0.787	0.71	1.074
Sample_MC19_2	0.03	0.039	2.222	0.962	1.215	0.324	0	0.76	0.053	1.657	1.37	0.9	0.75	0.469	0.51	0.397
Sample_MC19_3	0.86	0.723	0.512	2.631	0.402	0.39	0.706	0.75	0.783	1.161	1.86	0.66	0.06	0.41	0.94	0.528
Sample_MC20_1	2.06	0.719	1.095	1.449	0.681	0.109	0.595	0.63	1.478	0.643	1.4	0.78	0.57	0.581	0.51	0.291
Sample_MC20_2	0.75	0.441	1.151	0.74	0.521	0.82	0.748	0.89	0.214	0.837	1.18	1.19	0.54	0.827	0.71	0.67
Sample_MC20_3	0.78	0.752	0.562	1.477	0.599	2.985	0.768	0.61	0.159	0.549	0.96	0.68	0.26	0.323	0.34	0.76
Sample_MC21_1	2.77	1.09	0.543	0.646	1.774	0.261	0.797	1.32	1.212	0.389	0.38	0.8	1.91	0.795	0.36	0.366
Sample_MC21_2	2.68	1.717	0.411	1.726	1.357	0.822	0.663	0.88	0.253	0.354	0.38	0.82	1.46	0.217	0.81	1.111

Sample_MC21_3	1.36	1.594	0.574	1.62	0.844	0.929	0.532	0.38	2.481	0.946	0.65	0.79	0.17	0.924	0.84	0.468
Sample_MC22_1	3.22	2.107	0.715	0.516	2.104	1.21	0.485	1	2.696	0.366	0.29	0.52	1.79	0.622	0.32	0.35
Sample_MC22_2	2.48	2.139	1.011	0.431	1.355	1.003	0.583	0.79	2.119	0.172	0.32	0.47	1.61	0.903	0.8	0.247
Sample_MC22_3	1.35	2.599	1.255	0.968	0.082	2.102	0.817	0.78	2.212	0.221	0.27	0.53	0.61	1.151	0.88	0.323
Sample_MC23_1	1.73	0.455	1.612	1.149	0.496	0.52	0.548	0.4	0.878	0.369	0.25	0.62	0.71	1.213	0.48	0.518
Sample_MC23_2	0.95	0.202	1.076	0.758	0.431	0.054	0.512	1.07	0.057	1.043	0.53	0.85	0.12	0.619	0.34	0.863
Sample_MC23_3	1.44	0.742	1.385	1.738	0.351	1.534	0.432	0.66	0.993	0.128	0.86	0.49	0.11	0.312	0.89	0.418
Sample_MC24_1	2.86	1.67	0.559	0.615	2.183	1.359	0.548	0.94	0.713	0.7	0.92	0.77	0.91	0.323	0.76	0.619
Sample_MC24_2	0.93	0.823	0.663	0.36	0.838	0.646	0.546	0.64	0.193	3.275	0.76	0.9	0.28	0.193	0.56	0.763
Sample_MC24_3	0.64	0.233	0.287	0.586	0.338	1.158	0.442	0.33	0.138	1.051	0.78	0.63	0.13	0.065	0.34	1.074
Sample_MC25_1	0.67	1.77	0.708	1	1.522	1.712	0.437	0.9	0.353	0.884	0.71	1.03	0.35	0.423	0.79	1.108
Sample_MC25_2	1.37	1.257	0.705	1.371	1.628	1.25	0.655	0.94	1.359	1.144	0.52	1.13	0.8	0.202	0.93	0.702
Sample_MC25_3	1.27	1.067	0.957	1.927	1.806	1.231	0.504	1	1.978	0.223	0.54	0.7	0.23	0.344	0.57	0.595
Sample_MC26_1	0.4	1.418	2.224	1.718	0.547	0.35	0	0.43	0	0.108	1	0.5	0.27	1.482	0.72	0.324
Sample_MC26_2	0.24	0.484	3.501	2.123	0.958	0.893	0	0.68	0	0.062	0.36	0.32	0.55	0.841	0.75	0.187
Sample_MC26_3	1.34	1.287	1.329	1.833	0.456	1.265	1.172	0.41	0	0.104	0.54	0.59	0.16	0.34	0.15	0.596
Sample_MC27_1	3.91	2.309	1.412	1.364	1.745	0.741	0.718	0.48	1.349	0.309	0.52	0.53	1.13	1.747	0.59	0.519
Sample_MC27_2	2.72	3.772	1.163	0.314	1.971	0.944	0.6	1.03	0.862	0.041	0.07	0.61	2.05	1.357	0.44	0.45
Sample_MC27_3	0.17	0.114	1.438	0.555	0.794	0.06	18.32	0.54	0.063	0	0	0.28	2	0.552	0.05	0
Sample_MC28_1	1.3	2.297	1.372	0.399	1.827	1.136	0.854	0.92	1.558	0	0.05	0.43	1.79	1.098	0.6	0.131
Sample_MC28_2	1.68	3.372	1.29	0.359	0.739	0.72	1.066	1.2	0.191	0	0.19	0.69	1.47	0.951	0.43	0.164
Sample_MC28_3	0.99	0.751	0.918	0.702	0.658	0.802	0.452	0.79	3.037	0	0.09	0	1.03	2.091	0.62	0.156
Sample_MC29_1	0	1.673	0.332	1.942	1.517	1.85	1.612	0.91	0	1.183	1.65	0.56	0.5	0.226	0.33	0.58
Sample_MC29_2	0.03	0.261	0.962	0.668	1.058	0.823	0.726	0.85	0	1.457	1.58	0.5	0.55	0.999	0.62	0.792
Sample_MC29_3	0	0	0.748	0.215	0.987	0.293	0.586	0.83	0	0.232	2.3	0.54	0.16	0.51	0.53	0.308
Sample_MC30_1	3.04	0.354	0.382	2.197	1.565	0.862	0.53	0.87	0.107	0.595	0.77	0.89	2.19	0.429	0.64	0.501
Sample_MC30_2	1.43	0.164	0.51	0.714	0.574	0.234	0.68	1	0.076	0.491	0.28	0.45	2.17	0.31	0.42	0.579
Sample_MC30_3	0.51	0.108	0.39	1.491	0.871	0.527	0.706	0.41	0.179	0.825	0.99	0.48	0.23	0.344	0.69	0.109
Sample_MC31_1	1.22	2.146	1.971	2.549	0.296	0.558	0.54	0.5	0.829	0.08	0.68	0.62	0.18	0.457	0.54	0.694
Sample_MC31_2	2.26	1.901	1.683	2.375	0.473	1.113	0.537	0.87	1.347	0.094	0.55	0.64	0.37	1.628	0.51	0.53
Sample_MC31_3	1	0.968	1.341	0.828	0.844	0.647	0.424	0.79	1.522	0	0.16	0.35	0.23	1.339	0.27	0.177
Sample_MC32_1	2.99	3.618	1.299	0.759	0.641	1.405	0.734	0.81	2.091	0.588	0.42	0.57	0.44	0.485	0.9	0.297
Sample_MC32_2	2.62	2.957	1.205	0.546	0.806	1.653	0.853	1.2	2.921	0.08	0.08	0.51	1.28	0.906	0.74	0.253
Sample_MC32_3	1.55	1.353	1.388	0.614	0.263	0.811	0.514	0.64	2.965	0.064	0.21	0.49	0.15	0.738	0.32	0.101
Sample_MC33_1	0	0.28	0.607	0.632	1.768	0.315	0.473	0.94	0	0.649	0.37	0.38	0.25	0.43	0.3	1.228
Sample_MC33_2	0	0.087	0.714	0.111	0.185	0.369	0	0.7	0	0.715	0.7	0.54	0.08	0.313	0.33	1.067
Sample_MC33_3	0.19	0.062	0.895	0.656	1.546	0.614	0.751	0.49	0	0.066	1.19	0.6	0.28	0.777	0.34	0.356
Sample_MC34_1	1.72	1.71	1.579	1.148	0.52	1.409	0.485	0.92	1.244	0.079	0.42	0	0.89	0.904	1.1	0.19

Sample_MC34_2	1.77	0.607	1.336	0.958	0.728	0.059	0.73	1.35	2.123	0.103	0.39	0.36	1.28	0.998	0.39	0.192
Sample_MC34_3	1.79	0.633	1.399	0.493	0.095	0.605	0.571	0.71	2.371	0	0.24	0	0.88	0.922	0.78	0.074
Sample_MC35_1	1.43	0.473	2.134	0.984	0.928	0.858	0.463	0.55	0.222	1.824	1.42	0.57	0.37	0.438	0.52	0.518
Sample_MC35_2	0.65	0.277	1.154	0.697	0.982	1.076	1.603	0.83	0.322	0.602	1.51	0.49	0.27	0.427	0.41	0.967
Sample_MC35_3	0.45	0.094	1.551	1.021	1.132	0.879	3.289	0.61	0.671	0.045	0.75	0.38	0.13	0.464	0.38	0.308
Sample_MC36_1	2.63	1.244	0.527	2.256	1.21	1.675	0.458	0.53	0.398	1.755	1.33	0.69	0.25	0.25	0.92	0.495
Sample_MC36_2	1.68	0.419	1.076	2.646	1.611	0.663	0.64	1.17	0.152	0.436	1.25	0.78	0.36	0.51	1.01	0.369
Sample_MC36_3	0	0	0.362	0	2.694	0	1.052	0	0	0	0	0.31	0	0.135	0	0.023
Sample_MC37_1	0.17	0.603	1.132	1.162	1.127	1.569	0	0.63	0	1.264	0.46	0.44	0.11	0.615	0.31	0.413
Sample_MC37_2	0.8	0.264	1.402	1.06	1.003	0.308	0.484	1.1	0.082	0.309	0.64	0.54	0.23	0.665	0.61	0.287
Sample_MC37_3	0.27	0.142	0.834	0	1.122	0.307	0.972	0.06	0.289	0	0.25	0.74	0.39	0.654	0.7	0.561
Sample_MC38_1	2.22	0.174	0.959	0.673	1.691	1.453	0.478	1.18	0.631	0.204	0.6	0.44	1.93	0.775	0.48	0.461
Sample_MC38_2	2.16	0	0.828	1.296	1.929	0.761	0.642	0.85	0.238	0.474	0.84	0.44	1.49	0.766	0.7	0.533
Sample_MC38_3	0.08	0	0.086	0.109	0.984	0.111	2.673	0.72	0.085	0	0	0	0.84	0.221	0.05	0
Sample_ME01_1	0.4	0.62	1.028	0.701	1.185	0.34	0.494	0.66	0.191	1.944	0.81	1.19	0.36	0.193	0.32	0.912
Sample_ME01_2	1.24	1.674	0.599	0.858	0.506	0.473	0.782	0.51	0.168	0.413	1.06	1.11	0.45	0.086	0.81	0.806
Sample_ME01_3	1.72	0.376	0.769	1.043	0.457	1.326	1.041	0.91	2.937	0.03	0.5	0.53	0.45	1.201	0.63	0.133
Sample_ME02_1	0.47	0.412	0.689	0.404	1.014	0.523	0.584	0.56	0.331	3.307	1.38	0.52	0.11	0.124	0.78	0.81
Sample_ME02_2	0	0.424	0.599	0.253	0.258	0.363	0.518	0.29	0	3.067	1.63	0.88	0.09	0.121	0.47	1.383
Sample_ME02_3	0.06	0	0.647	0.869	0.911	0.667	0.619	1.01	1.275	0.229	1.24	0.65	0.45	0.574	0.76	0.557
Sample_ME03_1	2.17	0.875	0.38	0.367	1.171	0.803	0.539	0.51	0.683	1.43	0.67	0.57	0.75	0.336	0.57	1.094
Sample_ME03_2	1.06	0.624	0.304	0.571	0.41	0.307	0	0.44	0.573	2.078	0.78	0.68	0.39	0.237	0.45	1.227
Sample_ME03_3	0.66	0.039	0.904	0.069	0.613	0.149	1.222	0.91	1.063	1.92	2.09	0.63	0.16	0.179	0.39	0.679
Sample_ME04_1	2.65	0.215	0.925	0.772	1.593	1.393	0.418	1.12	0.787	0.804	0.26	0.52	1.96	0.372	0.61	0.676
Sample_ME04_2	3.64	0.773	0.352	2.028	0.451	0.766	0.63	0.75	0.405	1.774	0.75	0.75	0.38	0.523	0.3	0.72
Sample_ME04_3	1.6	0.271	0.694	0.226	0.673	0.869	0.709	1.15	3.172	0.04	0.81	0.6	0.21	0.285	0.29	0.262
Sample_ME05_1	2.25	0.133	0.517	0.939	0.778	0.967	0.793	0.83	1.001	3.328	0.47	0.54	0.56	0.932	0.91	0.462
Sample_ME05_2	1.59	0.226	0.31	0.997	0.397	0.954	0.675	0.6	0.26	0.388	1.26	0.66	0.25	0.205	0.3	1.099
Sample_ME05_3	1.57	0	0.831	1.242	2.936	0.103	0.421	0.82	5.729	0	0.16	0.4	0.61	0.482	0.77	0.726
Sample_ME06_1	2.78	1.639	0.886	0.827	0.814	1.228	0.432	0.82	1.859	0.157	0.24	0.53	1.62	2.02	0.67	0.609
Sample_ME06_2	2.07	0.986	0.6	1.293	0.596	0.966	0.515	0.48	0.257	0.892	1.15	1.02	0.96	0.432	1.23	0.456
Sample_ME06_3	1.59	0.629	0.78	0.573	1.203	1.28	0.634	0.83	0.896	0	0.63	0.64	0.57	0.629	0.81	0.556
Sample_ME08_1	0.17	0.121	1.422	0.908	0.508	0.293	0	0.59	0.153	1.404	0.79	0.53	0.17	0.461	0.31	0.445
Sample_ME08_2	0.35	0	1.197	2.136	0.14	0.174	0.455	0.84	0.577	2.856	1.24	0.47	0.19	0.582	0.23	0.634
Sample_ME08_3	0.25	0.148	1.28	0.642	0.985	0.501	0.404	0.72	1.613	0	0.36	0.33	0.84	1.022	0.42	0.16
Sample_ME09_1	1.17	0.665	0.598	0.591	1.093	0.361	0	1.16	0	2.232	1.14	0.58	0.23	0.105	0.2	1.079
Sample_ME09_2	0	0.357	0.57	0	0.103	0.183	0	0.53	0	1.684	1.61	0.98	0.07	0.224	0.17	1.24
Sample_ME09_3	2.14	0	0.618	1.082	1.043	1.371	0.755	0.84	0.122	1.012	1.35	0.49	0.38	0.359	0.33	0.448

Sample_ME10_1	0	0.179	0.5	0.213	0.345	0.12	0	0.62	0.053	2.044	2.19	0.72	0.08	0.275	0.38	0.605
Sample_ME10_2	0	0.333	0.308	0.347	0.167	0.221	0.572	0.57	0	0.772	1.66	0.81	0.07	0.091	0.1	1.437
Sample_ME10_3	0.74	0.396	1.732	4.482	0.763	0.722	0.478	0.66	0.076	0.332	1.17	1	0.07	0.199	0.43	0.226
Sample_ME11_1	2.34	2.719	1.325	1.323	1.175	1.056	0	0.73	0.715	0.552	0.63	0.64	0.49	2.089	0.79	0.684
Sample_ME11_2	0.57	2.222	1.036	1.1	0.412	0.396	0.567	0.73	0.065	0.577	0.7	1.05	0.16	0.244	0.26	1.642
Sample_ME11_3	1.2	0.323	0.908	0.47	2.008	0.118	1.034	0.95	2.35	0.267	0.54	1.42	1.02	1.534	0.63	0.153
Sample_ME12_1	2.16	0.59	0.803	1.162	1.14	0.965	0.451	0.82	1.561	0.579	0.57	0.56	0.99	0.353	0.66	0.451
Sample_ME12_2	3.1	1.85	0.955	1.244	0.727	0.648	0.52	0.67	2.037	1.07	0.71	0.98	0.3	0.142	0.65	0.893
Sample_ME12_3	1.36	0.373	1.12	1.545	0.854	0.318	0.492	0.73	2.936	0.382	1.35	0.62	0.14	1.093	0.28	0.284
Sample_ME14_1	1.68	0.624	0.511	0.943	1.157	0.76	0.455	0.59	0	0.954	1.18	0.78	2.76	0.525	0.69	0.467
Sample_ME14_2	0	0.234	0.397	1.087	0.197	0.044	0.543	0.59	0	0.695	1.13	1.58	0	0.18	0.28	0.825
Sample_ME14_3	1.54	0.635	1.887	3.216	0.157	1.729	0.543	0.59	0	0.143	1.3	0.49	0.1	0.15	0.49	0.324
Sample_ME15_1	1.79	2.845	1.164	0.386	1.236	1.814	0.528	0.87	3.124	0.099	0.1	1	1.37	1.65	0.53	0.225
Sample_ME15_2	2.51	4.306	0.488	0.465	0.467	0.444	0.538	0.65	0.268	0.625	0.24	1.09	0.94	0.655	0.65	0.592
Sample_ME15_3	1.97	0.033	1.053	1.197	1.216	0.266	0.428	0.8	1.905	0	0.07	0.43	3.72	0.594	0.63	0.443
Sample_ME16_1	0.43	0.468	0.863	0.2	0.733	0.321	0.537	0.68	0.062	2.25	0.86	1.8	0.15	0.147	0.26	0.63
Sample_ME16_2	0.72	0.439	0.652	0.58	0.461	0.246	0.51	0.46	0.118	2.963	1.52	0.58	0.1	0.063	0.44	1.12
Sample_ME16_3	1.65	1.363	1.105	1.168	2.063	0.983	7.463	1.08	0.964	0.813	0.42	0.43	0.39	0.583	0.39	0.229
Sample_ME17_1	0.86	1.015	1.124	0.231	0.322	0.9	0.618	1.8	0.247	0	0.03	0	1.98	1.137	0.15	0.051
Sample_ME17_2	1.92	2.79	1.3	0.536	0.607	1.148	0.667	0.95	1.17	0.051	0.23	0.45	1.75	0.771	0.5	0.103
Sample_ME17_3	0.05	0	0.67	0	1.097	0.291	1.688	0.4	3.619	0.499	1.17	1.45	1.87	0.231	0.34	0.196
Sample_ME18_1	2.91	1.887	1.345	1.316	0.661	1.736	0.66	0.44	1.14	0.875	0.61	0.7	0.32	0.367	0.38	0.415
Sample_ME18_2	1.98	1.205	1.014	1.287	0.452	0.809	0.497	0.87	0.799	1.057	1.18	0.79	0.19	0.25	0.55	0.584
Sample_ME18_3	1.86	1.228	1.455	1.364	1.215	1.359	0.856	1.21	4.312	0.152	0.29	0.63	0.35	0.615	0.27	0.423
Sample_ME19_1	2.81	2.167	1.071	1.041	0.841	0.387	0.859	0.65	1.28	0.402	0.91	0.78	0.93	0.974	0.6	0.78
Sample_ME19_2	2.3	1.526	1.055	1.024	0.338	0.638	0.647	0.35	0.354	0.779	1.08	0.81	0.2	0.401	0.32	0.782
Sample_ME19_3	1.73	1.453	1.159	1.266	0.05	0.506	1.367	1.21	1.862	0.335	0.82	0.48	0.21	0.59	0.4	0.198
Sample_ME20_1	2.02	1.383	1.134	0.854	0.947	1.553	0.739	0.7	0.212	1.784	0.6	0.64	0.35	0.383	0.49	0.851
Sample_ME20_2	0.93	0.368	0.572	0.708	0.187	0.433	0.519	0.58	0.035	1.683	0.96	0.72	0.2	0.463	0.31	0.751
Sample_ME20_3	1.62	1.953	1.445	0.918	1.268	1.915	0.594	0.85	0.251	0.109	0.32	0.77	0.15	0.512	1.11	0.125
Sample_ME21_1	2.13	1.735	2.176	0.779	0.906	2.114	0.577	0.92	0	0.306	1.07	0.59	0.5	0.473	0.6	0.59
Sample_ME21_2	0.08	0.195	0.945	0.806	0.223	0.904	0.662	0.58	0	1.312	0.95	0.65	0.11	0.389	0.3	1.236
Sample_ME21_3	0.6	0.037	1.002	1.54	1.209	0.591	1.405	0.81	0	0.093	0.37	0.76	0.14	0.106	0.18	0.609
Sample_ME22_1	1.83	0.384	1.406	0.63	1.012	0.17	0.594	0.77	0	0.816	0.59	0	1.35	0.732	0.8	1.087
Sample_ME22_2	1.94	0.986	0.637	1.367	0.213	0.672	0.48	0.78	0.84	1.38	1.7	0.55	0.37	0.705	0.37	0.977
Sample_ME22_3	1.26	0.368	0.67	1.837	0.81	1.67	0.423	1.03	1.846	0.413	1.3	0.47	0.73	0.997	0.45	0.626
Sample_ME23_1	1.45	2.593	0.821	0.596	0.862	1.091	0.46	1.12	0.214	0.452	0.48	0.65	0.44	0.635	0.87	0.377
Sample_ME23_2	1.9	1.143	0.548	1.055	0.187	0.987	0.495	0.7	0.245	0.99	0.75	0.52	0.36	0.307	0.84	0.612

Sample_ME23_3	1.18	0.33	1.255	2.14	0.27	0.365	2.692	0.62	2.36	0.204	0.43	0.44	0.46	0.644	0.35	0.169
Sample_ME24_1	1.15	0.721	0.561	0.76	1.363	0.913	0.564	0.94	0	2.121	0.63	0.91	2.01	0.253	0.53	0.707
Sample_ME24_2	2.19	1.124	0.464	1.14	0.85	0.698	0.489	1.06	0	2.986	0.4	1.19	1.01	0.116	0.9	0.68
Sample_ME24_3	0.97	1.264	1.088	0.815	1.206	0.75	0.774	0.74	0	0.241	0.24	0.85	0.5	1.298	0.84	0.06
Sample_ME25_1	1.84	1.215	0.92	0.473	0.913	0.836	0.523	0.7	0.34	1.06	0.48	0.95	1.22	1.066	0.72	0.833
Sample_ME25_2	1.81	0.964	0.93	1.262	0.494	0.88	0.599	0.61	0.178	0.487	1.09	0.86	0.66	0.246	0.48	0.502
Sample_ME25_3	1.73	2.174	1.009	0.863	0	1.078	3.607	0.67	1.03	0	0.65	0.76	0.25	0.772	0.79	0.077
Sample_ME26_1	3.81	1.788	0.601	0.331	1.33	1.336	0.703	1.12	0.461	0.906	0.61	0.74	2	0.197	0.91	0.405
Sample_ME26_2	2.52	1.045	0.461	0.721	0.745	0.263	0.857	0.59	0.093	1.323	0.83	0.93	0.38	0.1	0.61	0.441
Sample_ME26_3	1	2.226	0.541	1.381	1.818	0.667	0.475	1.16	1.587	1.122	0.35	0.56	0.51	0.345	0.22	0.383
Sample_ME27_1	0	0.032	0.888	0.697	0.446	0.083	0.446	0.15	0	0.293	0.23	0.89	0.18	0.196	0.48	1.272
Sample_ME27_2	0	0.065	1.125	0.535	0.44	0.063	0.563	0.2	0	2.887	0.82	0.46	0.07	0.125	0.25	0.377
Sample_ME27_3	0.09	0.502	0.677	2.619	1.018	1.285	1.479	0.48	0.284	0.178	0.72	0.39	0.32	0.495	0.92	0.156
Sample_ME28_1	2.19	2.91	1.252	0.379	1.356	1.563	0.646	1.25	0.168	0.168	0.19	0.79	1.05	1.5	0.7	0.442
Sample_ME28_2	2.26	2.718	1.582	0.554	0.814	1.661	0.509	0.67	0.094	0.106	0.18	0.45	0.49	0.333	0.65	0.376
Sample_ME28_3	1.47	2.058	2.154	1.622	0.085	2.122	1.026	0.5	0.431	0.053	0.26	0.37	0.59	1.308	1.08	0.135
Sample_ME29_1	0.21	0.435	1.097	0.844	0.813	0.108	0.487	0.92	0.043	2.055	1.11	0.68	0.26	0.349	0.3	1.162
Sample_ME29_2	0	0.468	0.456	0.465	0.243	0.978	0	0.67	0	1.327	0.8	1.18	0.13	0.093	0.18	1.111
Sample_ME29_3	1.61	1.12	0.827	0.778	1.311	1.594	0.869	0.75	0.23	2.116	0.7	0.71	0.22	0.199	0.31	0.505
Sample_ME30_1	1.39	2.051	0.782	1.025	2.31	1.121	0.745	1	0.623	0.514	0.58	0.53	0.39	0.51	0.68	0.625
Sample_ME30_2	2	0.579	0.516	2	0.553	1.232	0.541	0.44	0.54	0.343	1.46	0.54	0.31	0.795	0.69	0.443
Sample_ME30_3	2.16	1.762	1.202	1.531	1.24	1.351	0.833	0.74	0.691	0.102	0.59	0.57	0.43	0.486	0.84	0.312
Sample_ME31_1	1.37	0.636	0.612	0.706	0.539	1.045	0	0.6	0	1.737	0.87	0.94	0.11	0.65	0.68	1.293
Sample_ME31_2	0.19	0.984	0.73	0.892	0.203	0.561	0.52	0.68	0	1.641	1.01	0.8	0.25	0.312	0.39	1.443
Sample_ME31_3	0	0.594	0.679	0.582	0.658	0.536	0.383	1.04	0	0.477	0.83	1.8	0.22	0.305	0.1	0.756
Sample_ME32_1	1.89	2.461	1.895	0.693	0.405	0.311	0.487	0.9	0	0.238	0.38	0.42	0.63	0.844	0.34	0.395
Sample_ME32_2	2.55	3.633	1.942	0.683	0.371	2.052	0.533	0.5	0	0.068	0.55	0.45	0.29	0.45	1.38	0.458
Sample_ME32_3	0.37	0.125	1.091	0.937	0.956	0.569	0.605	1.08	0.08	0.252	0.19	0.28	0.8	0.532	0.15	0.251
Sample_ME33_1	2.49	1.9	1.518	0.295	0.999	1.591	0.822	0.55	0.253	0.051	0.31	0.61	0.98	0.565	0.57	0.315
Sample_ME33_2	2.26	1.983	1.295	0.921	0.206	1.165	1.236	0.71	0.494	0.333	0.4	0.59	1.31	0.58	0.48	0.358
Sample_ME33_3	2.27	1.112	1.164	2.11	0.594	1.322	1.671	0.52	1.159	0.048	0.46	0	0.37	0.266	0.4	0.716
Sample_ME34_1	1.21	0.526	1.163	0.543	1.432	0.344	0	0.62	0.036	1.708	0.74	0.77	0.44	1.008	0.59	0.916
Sample_ME34_2	0.1	0.531	0.534	0.876	0.405	0.684	0	0.5	0	2.458	1.34	0.77	0.09	0.137	0.39	0.506
Sample_ME34_3	1.85	0.096	0.545	2.309	0.896	0.263	0.934	0.63	3.715	0.092	1.27	0.54	0.08	0.451	0.58	0.309
Sample_ME35_1	1.31	1.264	1.835	0.375	2.125	0.644	0.443	1.34	1.44	0	0.17	0.57	2.37	1.251	1.58	0.188
Sample_ME35_2	2.49	1.647	1.036	2.292	0.77	0.57	0	0.66	1.485	0.217	0.72	0.99	0.99	0.417	0.77	0.19
Sample_ME35_3	1.28	1.45	1.484	0.286	0.325	1.04	1.239	1.76	0	0.029	0.09	0	0.31	0.848	0.27	0.144
Sample_ME36_1	0.38	0.264	0.305	1.357	0.983	1.261	0	0.52	0	2.712	1.03	0	0.59	0.233	0.8	0.775

Sample_ME36_2	0.38	0.28	0.425	0.062	0.433	0.169	0.489	1	0	0.4	0.81	0.52	0.64	0.337	0.18	1.194
Sample_ME36_3	1.18	0	1.537	1.251	0.795	0.166	0.856	0.85	0	0.718	1.39	0.58	0.45	0.183	0.28	0.527
Sample_ME37_1	0	0	0.467	0.076	0.194	0.222	0	0.74	0	1.147	0.79	0.69	0.2	0.078	0.34	1.399
Sample_ME37_2	0	0	0.164	0.677	0	0	0	0.69	0	1.79	1.89	0.59	0.22	0.191	0.16	0.626
Sample_ME37_3	0.41	0	0.93	0.734	1.614	1.728	0.682	0.37	0.258	0.199	1.83	0.75	0.07	0.647	0.26	0.362
Sample_ME38_1	0.55	0.712	1.181	0.619	1.753	1.666	0.874	1.63	0.114	0.141	0.06	0.57	1.14	1.264	0.41	0.274
Sample_ME38_2	1.73	0.195	0.408	2.858	0.927	1.293	0.806	0.49	0.211	0.313	0.4	0.55	0.81	0.58	0.78	0.505
Sample_ME38_3	1.19	1.104	1.954	0.642	0.142	0.056	10.46	0.95	0.584	0	0	0	1.15	1.056	0.09	0.099

Supplementary table 5. Significant differential gut bioactive metabolites between probiotic and control groups

Bioactive metabolite	Gestation 100d			Gestation 113d			Lactation 23d		
	Control_mean	Probiotic_mean	P_value, Wilcoxon Test	Control_mean	Probiotic_mean	P_value, Wilcoxon Test	Control_mean	Probiotic_mean	P_value, Wilcoxon Test
hydrocinnamic acid	0.0002998	0.00027637	0.43952841	0.00023482	0.00038839	0.00165307	0.00023576	0.00020268	0.51997278
creatine	0.00058456	0.00067091	0.17830936	0.00055634	0.00070005	0.00657654	0.00054982	0.00049157	0.48039857
chenodeoxycholate	0.0003544	0.00039472	0.52466373	0.00035486	0.0004531	0.04710176	0.00034944	0.00032864	0.90802314
arachidonic acid	0.00060256	0.00066576	0.08021719	0.00061372	0.00066682	0.03778511	0.00060702	0.00059916	0.88685213
ceramide	0.00011093	0.00011228	0.76925473	0.00011084	0.00011655	0.03778511	0.00010809	0.00010539	0.42834468
C18:1 cholesterol esters	0.00010475	0.00010639	0.46433986	0.00010509	0.00010953	0.04306395	0.00010437	0.00010186	0.52583784
palmitoyl glycerol	7.03E-05	7.18E-05	0.08021719	7.15E-05	7.27E-05	0.04710176	7.22E-05	7.13E-05	0.23423349
C18:0 Sphingomyelin	2.54E-05	2.66E-05	0.08021719	2.61E-05	2.71E-05	0.05	2.65E-05	2.55E-05	0.23244763
phenylacetate	5.99E-05	5.68E-05	0.65162568	6.21E-05	5.07E-05	0.00037123	5.89E-05	6.66E-05	0.13311938
pyridoxamine	3.68E-05	3.17E-05	0.08021719	3.60E-05	2.93E-05	0.00233	3.47E-05	3.60E-05	0.7574228

C20:4 carnitine	0.0001161	0.00011419	0.65906773	0.0001166	0.00010736	0.03552319	0.00011771	0.00012297	0.29035197
sebacate	0.00011746	0.00011379	0.40526427	0.00011858	0.00010281	0.01933474	0.00011822	0.00012846	0.20038787
azelate	0.00031179	0.00028885	0.11206138	0.00029756	0.00027507	0.04823555	0.00029991	0.00032744	0.16154956
lithocholic acid	0.00100045	0.00088731	0.1899621	0.00095342	0.000811	0.04823555	0.00092408	0.00111039	0.13311938
glutamate	0.00383256	0.00363322	0.1899621	0.0038021	0.00351021	0.02678824	0.00389854	0.00397403	0.86507715
undecanedionate	5.85E-05	5.12E-05	0.10926904	5.43E-05	5.01E-05	0.32340055	5.01E-05	6.32E-05	0.03311034

Supplementary table 6. Distribution of genes encoding carbohydrate-active enzymes (CAZymes) in different species genome bins (SGBs) in the sow fecal microbiota

SGB_ID	Auxiliary Activities family (AA)	Carbohydrate-Binding Module family (CBM)	Carbohydrate Esterase family (CE)	Glycoside Hydrolase family (GH)	GlycosylTransferase family (GT)	Polysaccharide lyase family (PL)
SGB_47	0	0	8	57	23	0
SGB_28	0	3	7	39	13	0
SGB_92	0	6	9	41	27	0
SGB_11	0	10	9	60	15	0
SGB_89	2	9	25	178	30	8
SGB_44	0	1	7	9	13	0
SGB_2	0	0	2	2	5	0
SGB_80	1	0	1	1	9	0
SGB_6	0	3	9	21	23	0
SGB_42	0	2	5	7	10	0
SGB_65	0	4	17	62	19	0
SGB_3	0	4	6	36	15	0
SGB_200	0	0	6	27	25	0
SGB_29	0	1	4	8	8	0
SGB_32	0	3	7	23	5	1
SGB_140	0	2	5	23	26	0
SGB_137	3	2	13	40	30	0
SGB_288	0	8	5	31	32	0
SGB_180	0	5	7	35	11	0
SGB_93	0	4	7	53	15	1
SGB_208	0	6	17	96	9	3
SGB_121	0	2	2	24	4	0
SGB_326	0	4	6	23	7	2
SGB_3	0	1	10	62	20	4
SGB_88	0	1	2	9	10	0
SGB_58	0	24	9	48	35	7
SGB_319	0	2	6	47	24	0
SGB_306	0	14	7	66	12	0
SGB_298	0	2	5	38	10	0
SGB_153	1	0	4	17	12	0
SGB_41	1	0	1	2	9	0
SGB_86	0	3	15	80	23	7

SGB_183	0	1	6	22	12	0
SGB_270	0	3	12	86	29	0
SGB_123	1	0	1	2	17	0
SGB_209	0	6	2	28	15	0
SGB_257	1	2	5	23	13	0
SGB_193	0	5	7	27	35	0
SGB_241	0	5	13	66	13	0
SGB_303	0	1	15	67	15	2
SGB_28	0	3	28	79	19	4
SGB_65	0	4	23	80	38	6
SGB_320	0	1	8	16	11	0
SGB_67	0	2	4	11	5	0
SGB_296	0	5	15	67	31	0
SGB_279	0	2	4	20	9	1
SGB_132	1	0	2	3	17	0
SGB_114	1	1	6	6	14	0
SGB_239	1	2	14	84	14	1
SGB_154	0	1	5	22	4	2
SGB_161	0	0	9	21	6	2
SGB_66	0	9	7	37	32	0
SGB_243	1	0	12	26	4	2
SGB_126	0	4	8	20	18	6
SGB_4	0	1	3	6	16	0
SGB_156	0	1	0	2	8	0
SGB_229	0	5	7	16	23	0
SGB_159	0	1	2	2	10	0
SGB_250	0	3	5	18	18	0
SGB_63	0	7	15	60	25	0
SGB_95	0	2	5	18	27	0
SGB_151	0	1	9	57	16	3
SGB_181	0	2	7	53	8	0
SGB_312	0	2	6	20	29	1
SGB_205	0	0	4	15	19	0
SGB_166	0	1	3	1	4	0
SGB_2	0	1	5	10	11	0
SGB_27	0	1	5	21	23	0
SGB_222	0	1	9	56	20	1
SGB_19	0	36	13	68	10	6
SGB_73	0	4	9	26	29	6
SGB_332	0	2	9	55	12	0

SGB_48	1	0	1	2	14	0
SGB_203	0	8	7	62	17	0
SGB_215	1	2	7	11	12	0
SGB_294	0	3	7	51	15	0
SGB_62	0	3	16	92	29	0
SGB_148	0	1	5	15	9	0
SGB_64	0	2	3	18	12	0
SGB_130	0	4	12	73	22	5
SGB_264	1	7	8	30	21	0
SGB_60	0	1	8	48	13	0
SGB_122	0	4	8	32	23	3
SGB_317	0	2	6	36	8	0
SGB_133	0	2	11	31	28	0
SGB_277	0	3	6	13	15	0
SGB_112	0	1	6	16	5	0
SGB_147	0	2	2	13	13	0
SGB_72	0	7	12	81	34	1
SGB_155	0	0	6	20	13	3
SGB_254	0	5	8	21	20	0
SGB_46	0	2	5	78	28	1
SGB_34	1	5	3	35	24	0
SGB_43	0	34	17	62	14	5
SGB_267	0	2	3	15	10	0
SGB_105	0	1	3	12	10	0
SGB_197	1	4	8	47	12	0
SGB_81	0	5	12	61	31	0
SGB_96	0	5	22	84	39	9
SGB_295	0	5	7	37	10	1
SGB_232	0	2	7	38	10	0
SGB_248	0	6	6	29	17	0
SGB_220	0	2	10	35	33	0
SGB_274	0	5	13	56	10	0
SGB_189	0	4	19	78	33	2
SGB_157	1	1	3	10	22	0
SGB_230	0	2	11	34	25	0
SGB_146	0	4	6	16	5	0
SGB_92	0	0	2	7	8	0
SGB_304	0	9	12	97	29	0
SGB_185	0	0	2	10	10	0
SGB_251	0	2	4	25	7	1

SGB_271	1	2	10	28	10	0
SGB_120	0	0	1	3	7	0
SGB_61	0	1	8	10	5	0
SGB_261	0	1	12	25	16	0
SGB_337	2	0	0	0	4	0
SGB_278	0	6	24	86	32	19
SGB_101	0	4	9	48	15	1
SGB_310	0	5	6	24	8	0
SGB_284	3	0	0	0	6	0
SGB_339	1	0	1	3	17	0
SGB_196	0	1	11	9	19	0
SGB_187	0	5	11	68	13	0
SGB_311	0	2	4	9	14	0
SGB_223	0	1	8	39	28	0
SGB_79	0	2	4	21	22	0
SGB_131	0	4	3	33	15	0
SGB_36	0	3	2	15	7	0
SGB_262	0	5	10	32	11	0
SGB_57	0	2	1	7	18	0
SGB_290	1	1	3	3	18	0
SGB_235	0	4	5	38	13	0
SGB_49	1	1	8	53	15	0
SGB_190	0	3	12	70	28	11
SGB_134	0	8	22	103	32	4
SGB_216	0	1	18	50	17	0
SGB_186	0	8	3	27	16	0
SGB_23	0	4	12	59	23	0
SGB_29	0	0	3	0	2	0
SGB_198	0	7	7	39	14	0
SGB_128	0	2	8	18	14	0
SGB_313	2	7	12	77	11	1
SGB_13	0	2	2	11	5	0
SGB_212	1	3	8	35	8	1
SGB_265	0	4	5	50	18	0
SGB_127	0	5	7	33	9	0
SGB_31	0	1	4	12	10	0
SGB_201	0	3	8	114	26	0
SGB_167	0	1	1	1	7	0
SGB_314	1	6	15	46	25	0
SGB_210	2	8	12	102	14	1

SGB_266	0	3	3	21	10	0
SGB_149	0	0	12	33	17	0
SGB_219	0	3	2	26	13	0
SGB_22	0	1	6	12	17	0
SGB_244	0	2	14	45	25	4
SGB_35	0	1	2	5	10	0
SGB_98	0	1	6	29	32	0
SGB_194	0	3	5	42	25	0
SGB_173	0	15	12	93	28	0
SGB_53	0	3	11	18	14	5
SGB_30	0	14	20	120	46	18
SGB_233	0	1	26	52	11	0
SGB_160	0	3	9	25	30	0
SGB_199	0	2	8	10	13	0
SGB_106	0	1	3	8	17	0
SGB_75	0	1	4	8	11	0
SGB_217	0	5	6	24	22	0
SGB_1	0	5	10	53	15	0
SGB_276	0	1	2	3	9	0
SGB_263	0	1	10	43	8	0
SGB_135	0	4	7	61	9	0
SGB_289	0	7	19	148	22	5
SGB_16	0	0	2	16	8	0
SGB_119	0	3	9	22	13	0
SGB_10	0	9	8	37	14	0
SGB_164	0	0	2	12	24	0
SGB_140	0	0	9	51	23	1
SGB_103	0	8	23	162	65	9
SGB_14	0	6	13	85	35	0
SGB_234	0	4	18	73	26	1
SGB_253	0	3	8	32	16	2
SGB_45	0	6	20	88	47	3
SGB_25	0	5	10	60	20	0
SGB_184	0	3	20	89	42	7
SGB_33	0	1	8	83	25	0
SGB_69	0	4	7	22	8	1
SGB_240	0	2	12	82	15	2
SGB_74	0	4	20	93	31	12
SGB_12	0	33	19	60	9	7
SGB_286	0	0	4	73	21	7

SGB_321	0	3	8	48	29	1
SGB_280	0	0	9	28	6	0
SGB_54	0	11	21	71	24	1
SGB_305	0	1	11	17	30	4
SGB_9	1	19	10	41	28	0
SGB_200	0	2	13	67	13	0
SGB_204	2	1	1	7	17	0
SGB_307	0	11	5	46	11	2
SGB_325	0	3	11	48	21	0
SGB_111	0	0	1	3	13	0
SGB_335	0	0	2	1	6	0
SGB_246	0	4	6	42	12	0
SGB_76	0	4	13	61	13	0
SGB_255	0	4	12	49	28	4
SGB_109	0	1	2	5	10	0
SGB_91	0	9	6	57	24	0
SGB_258	0	1	11	57	24	0
SGB_38	0	0	1	4	15	0
SGB_104	0	2	2	7	21	0
SGB_18	0	2	4	19	10	0
SGB_21	0	1	1	3	8	0
SGB_259	1	0	1	3	13	0
SGB_144	1	0	2	13	16	0
SGB_272	0	1	5	18	4	0
SGB_169	0	5	24	108	29	11
SGB_336	0	1	5	38	13	0
SGB_236	0	0	5	19	16	0
SGB_331	0	1	4	25	12	1
SGB_108	0	2	11	46	26	6
SGB_327	0	0	6	51	23	0
SGB_315	1	1	2	10	26	0
SGB_256	1	9	23	187	21	5
SGB_89	0	0	1	2	8	0
SGB_136	0	0	7	19	27	4
SGB_177	0	0	2	2	11	0
SGB_20	0	2	5	28	14	0
SGB_172	0	8	8	57	16	1
SGB_301	0	4	14	65	24	2
SGB_71	0	45	21	82	14	5
SGB_224	0	10	16	84	27	0

SGB_328	0	4	6	70	19	0
SGB_162	0	2	3	6	17	0
SGB_55	0	0	8	42	14	0
SGB_139	0	1	3	7	11	0
SGB_116	1	7	7	28	12	0
SGB_165	0	2	7	64	25	0
SGB_268	1	2	6	30	9	0
SGB_324	0	8	4	15	8	0
SGB_107	0	0	9	42	23	0
SGB_40	0	8	10	75	19	0
SGB_300	7	0	6	7	11	0
SGB_171	0	16	11	145	55	2
SGB_338	2	0	0	0	5	0
SGB_94	0	6	4	29	15	0
SGB_287	0	19	6	35	12	3
SGB_141	0	3	6	23	24	1
SGB_158	0	1	5	12	11	0
SGB_228	0	0	1	2	12	0
SGB_83	0	1	2	7	6	0
SGB_245	0	3	5	49	17	0
SGB_42	0	1	5	24	33	0
SGB_285	0	3	9	17	31	0
SGB_163	0	2	6	16	9	0
SGB_330	0	1	5	18	5	0
SGB_192	1	0	1	2	24	0
SGB_150	0	4	8	29	26	0
SGB_118	2	0	0	0	4	0
SGB_90	0	0	10	44	27	1
SGB_283	0	1	8	30	20	0
SGB_124	0	5	5	30	27	0
SGB_82	0	3	4	49	30	1
SGB_11	0	3	28	94	33	5
SGB_176	0	6	13	85	28	0
SGB_17	0	5	11	39	13	0
SGB_226	0	1	6	11	15	0
SGB_179	1	4	17	48	15	0
SGB_170	0	6	20	79	21	2
SGB_59	0	6	5	41	14	0
SGB_138	1	3	4	14	5	0
SGB_178	1	0	12	29	9	3

SGB_50	1	4	11	30	16	0
SGB_100	0	4	7	18	12	0
SGB_231	0	2	8	43	16	0
SGB_5	0	4	15	39	17	3
SGB_80	0	2	2	23	23	0
SGB_97	0	5	7	34	9	0
SGB_47	0	9	24	79	32	3
SGB_293	0	0	2	0	4	0
SGB_85	0	1	3	11	12	0
SGB_282	1	0	3	10	19	0
SGB_214	0	1	2	1	5	0
SGB_117	0	0	2	1	4	0
SGB_227	0	18	8	44	30	7
SGB_269	0	0	8	11	10	0
SGB_318	0	4	8	28	24	0
SGB_323	0	3	13	43	19	3
SGB_238	0	1	1	6	6	0
SGB_142	0	3	6	50	35	0
SGB_221	0	4	12	49	15	0
SGB_297	0	6	19	65	20	0
SGB_213	0	3	13	61	26	0
SGB_32	0	0	6	16	27	0
SGB_316	0	7	16	97	29	5
SGB_129	0	4	4	15	11	0
SGB_247	0	0	5	36	8	1
SGB_207	0	5	7	25	3	1
SGB_242	0	0	1	3	16	0
SGB_152	0	1	4	12	10	0
SGB_87	0	2	5	1	12	0
SGB_7	0	1	3	4	16	0
SGB_99	0	3	15	68	33	8
SGB_260	0	5	6	50	26	0
SGB_84	0	2	6	17	4	0
SGB_70	0	0	3	1	6	0
SGB_322	0	0	1	1	5	0
SGB_334	0	8	12	49	12	1
SGB_174	0	8	8	65	21	0
SGB_115	0	4	10	23	28	0
SGB_56	0	4	8	58	25	1
SGB_68	0	0	3	10	14	0

SGB_309	0	2	12	30	29	0
SGB_191	0	2	4	20	12	0
SGB_202	0	2	11	28	12	0
SGB_37	0	1	3	5	7	0
SGB_329	0	11	12	121	30	1
SGB_188	0	9	7	79	26	0
SGB_168	0	4	20	65	20	0
SGB_145	0	5	17	78	23	1
SGB_113	0	4	10	66	15	0
SGB_206	1	1	10	24	15	1
SGB_218	0	0	7	34	14	8
SGB_78	0	1	3	11	18	0
SGB_24	0	1	3	1	5	0
SGB_302	0	3	6	41	10	0
SGB_26	0	2	6	20	27	0
SGB_237	0	3	10	56	11	0
SGB_273	0	0	2	1	4	0
SGB_281	0	1	2	27	8	0
SGB_211	0	0	4	35	13	0
SGB_308	0	3	5	19	7	0
SGB_110	0	4	12	34	15	0
SGB_52	0	0	1	5	10	0
SGB_333	0	2	9	55	13	0
SGB_51	0	0	5	5	21	0
SGB_143	0	3	17	65	21	0
SGB_175	0	0	2	1	4	0
SGB_299	0	5	24	146	22	1
Sum gene number	62	1204	2707	12727	5845	339

Supplementary table 7. Significant differential carbohydrate-active enzymes (CAZymes) between probiotic and control groups

CAZyme subfamily	Gestation 100d			Gestation 113d			Lactation 23d		
	Control_mean	Probiotic_mean	P_value, Wilcoxon Test	Control_mean	Probiotic_mean	P_value, Wilcoxon Test	Control_mean	Probiotic_mean	P_value, Wilcoxon Test
GT2_Glycos_transf_2	2952.55	3035.11	0.42	2877.65	3211.74	1.7E-08	2767.72	2775.85	0.85
GH2	1487.87	1465.54	0.49	1363.53	1515.79	0.00000017	1643.25	1356.84	1
GH92	207.09	238.80	0.7	202.12	301.98	0.0000047	197.12	184.54	1
GH78	400.47	445.77	0.085	415.37	508.77	0.00016	410.10	411.47	1
GH23	579.30	548.10	1	489.59	572.97	0.0000016	609.92	509.94	1
GH97	511.11	520.04	0.78	494.95	577.89	0.00000058	420.21	425.61	0.78
GT4	1595.26	1645.36	0.1	1581.19	1662.95	0.0043	1542.59	1539.22	1
GH20	396.94	405.32	0.17	372.48	445.71	0.017	481.16	357.69	1
GH13	263.05	259.39	0.64	213.59	282.98	0.014	301.25	216.22	1
PL1_2	101.44	109.13	1	90.30	149.14	0.000018	101.40	83.84	0.91
GH133	201.50	197.77	0.075	163.78	217.31	0.0000032	240.21	163.10	1
GH13_38	190.96	184.82	0.25	146.96	200.30	0.0000015	222.97	151.11	0.93
GH53	234.57	253.95	0.57	235.49	287.84	0.0018	192.91	189.84	0.69
GH13_8	189.78	187.97	0.071	153.57	204.44	0.000035	220.85	156.29	1
GT30	124.77	140.77	0.38	119.17	164.64	0.0000013	105.29	103.72	0.75
CE10	966.04	942.23	1	916.12	958.63	0.0000015	1031.58	912.35	1
CE7	130.69	129.73	0.23	106.65	148.82	0.00032	177.79	100.93	0.63
GH95	272.92	267.04	1	253.47	295.29	1.4E-08	257.85	257.59	1
GH43_10	262.39	245.22	0.29	231.07	272.82	0.0013	210.84	206.73	0.69
GH13_6	74.32	76.17	0.55	57.01	97.55	0.0063	114.82	48.16	0.6
GH28	439.98	408.32	1	397.03	437.46	7.3E-08	516.29	370.68	0.79
CBM32	198.95	176.51	1	163.38	203.23	0.0025	202.49	154.08	0.75
GH57	271.34	280.14	0.55	255.89	294.46	0.000024	223.66	227.42	0.83
GT3	132.17	148.21	0.23	130.74	168.60	0.00045	105.36	101.08	0.61
GH31	634.71	628.17	1	596.58	630.22	0.000029	689.50	599.36	1
GT83	66.40	78.55	1	64.61	97.21	0.000076	59.77	53.47	1
GH106	197.31	197.53	0.32	187.17	214.93	0.000015	246.08	183.77	1
CE8	246.65	220.40	1	215.02	241.31	2.2E-08	281.14	197.27	0.45
GH35	197.61	176.40	1	167.50	192.04	2.8E-09	221.98	163.54	1
GT9	164.09	171.83	0.34	155.14	179.38	0.00091	138.60	143.04	0.73

CBM20	62.60	62.92	0.38	52.11	75.94	0.043	80.37	47.86	1
GH13_10	69.13	70.65	1	50.39	73.66	0.024	83.27	50.70	1
GH146	185.46	169.68	0.33	152.97	175.59	0.038	190.64	155.21	1
GH128	48.54	41.42	1	28.21	50.42	0.049	48.74	28.57	1
PL1	70.32	65.07	0.93	60.55	82.50	0.00032	61.00	56.06	0.93
GH76	23.38	30.14	0.47	24.38	41.89	0.000075	24.95	24.70	1
GH50	25.84	34.33	0.12	28.60	46.07	0.00011	27.75	26.16	1
GH5_7	70.48	73.45	1	68.30	84.62	0.00029	57.88	55.87	1
GH5_21	63.21	60.36	1	56.34	71.23	0.00048	54.56	52.18	1
GT10	26.65	33.48	0.76	23.66	37.71	0.0025	15.79	13.42	0.76
GH89	69.73	76.79	0.2	70.16	83.43	0.0021	64.06	55.17	0.2
GH85	16.54	17.98	1	12.73	25.63	0.000035	14.22	11.68	1
PL10_1	57.97	55.97	1	48.63	61.37	0.034	47.52	40.94	0.39
GH84	41.29	48.98	0.1	39.32	51.41	0.00012	42.45	35.59	0.32
CBM62	50.57	40.63	0.85	35.14	47.21	0.002	53.17	37.83	1
GH105	301.65	276.54	0.34	271.34	283.34	0.024	307.88	256.66	1
GH30_5	56.10	47.01	0.73	40.06	51.70	0.0034	59.05	41.39	1
GH43_18	46.54	42.84	1	41.22	52.69	0.0011	42.59	40.13	1
GH123	78.18	81.73	0.12	79.22	89.32	0.022	98.76	76.86	1
GH148	40.41	43.58	0.16	41.04	50.93	0.0012	61.99	41.10	1
GH43_31	16.00	19.75	0.26	17.85	26.68	0.00061	19.04	17.88	1
GT27	15.60	19.00	0.3	15.61	24.43	0.00012	16.98	15.89	1
GT23	18.27	20.17	1	18.26	26.01	0.00013	19.83	16.19	1
GH5	13.58	18.01	0.09	15.19	22.80	0.00019	17.10	15.79	1
GH32	152.11	155.19	0.87	153.75	161.20	0.03	179.02	144.69	1
GH5_46	18.09	22.45	0.15	21.28	28.22	0.028	14.75	15.09	0.82
GH13_36	82.78	90.19	1	81.72	88.49	0.00071	71.63	83.89	1
GH13_3	17.81	17.10	0.59	14.53	20.43	0.0092	21.81	15.94	1
GT2_Glyco_tranf_2_4	4.61	6.27	1	4.67	10.54	0.000019	5.61	4.93	1
GH33	112.69	109.40	0.47	119.93	125.70	0.00019	142.57	105.95	0.47
GT2_Glyco_tranf_2_3	184.13	186.85	1	190.48	195.81	1	181.23	161.80	0.047
GT81	10.04	10.26	0.91	8.55	13.59	0.0013	7.62	6.74	0.76
PL33_2	7.03	8.77	1	8.25	12.99	0.005	9.44	9.01	1
GH110	36.22	38.25	0.15	37.81	42.13	0.0097	47.26	34.39	0.62
GH13_19	6.78	7.95	0.3	4.93	8.51	0.028	11.67	5.32	0.94
PL9	4.51	6.52	0.67	4.62	8.00	0.00033	4.54	2.73	0.79
GH5_36	2.36	3.82	0.13	2.67	5.49	0.00002	2.82	2.49	1
PL35	4.67	5.93	0.18	4.97	7.61	0.00061	5.31	4.46	1

GH5_19	6.41	8.10	0.33	7.11	9.35	0.029	8.74	8.22	1
CBM77	31.24	29.45	1	26.90	29.03	0.019	36.64	27.04	1
PL27	1.52	2.53	0.072	2.07	3.77	0.00054	1.97	2.23	1
GH99	2.99	3.43	1	2.68	4.25	0.00018	3.10	3.77	1
GH109	314.74	339.33	0.27	350.05	351.19	0.018	399.09	348.95	1
GH71	1.51	2.03	0.34	1.62	2.69	0.00063	1.80	1.80	1
GH43_8	1.10	1.02	1	0.82	1.58	6.6E-08	1.03	0.90	1
GH59	0.19	0.42	0.25	0.28	0.72	0.019	0.31	0.16	1
GH13_16	0.79	0.48	1	0.48	0.85	0.00092	1.01	0.58	1
GH19	1.01	0.94	0.2	1.85	1.94	0.000006	2.76	1.16	0.92
CBM57	16.36	17.62	0.28	20.73	20.50	0.0016	27.43	16.28	0.37
CBM37	1.19	1.34	0.16	1.15	0.89	0.0079	0.78	1.18	1
GT2_Glyco_tranf_2_2	1.04	1.01	1	0.79	0.29	0.016	0.79	0.48	1
GT46	1.30	1.35	1	1.58	1.08	0.00026	1.81	2.12	1
GT82	0.29	0.11	0.11	0.52	0.00	0.0000043	0.84	0.15	0.68
PL2_2	0.29	0.11	0.11	0.52	0.00	0.0000043	0.84	0.15	0.68
GH103	3.84	3.78	1	5.59	5.04	0.043	6.77	3.75	1
GT70	0.95	0.52	0.13	0.83	0.27	0.0016	0.37	0.26	0.83
GH137	15.03	14.53	0.58	19.14	18.42	0.00000091	27.28	14.67	0.38
GH129	15.90	18.82	0.22	21.48	20.75	0.0057	26.97	21.03	1
CE13	2.59	3.16	1	3.62	2.69	0.00026	2.16	4.24	1
GH139	6.54	6.45	0.5	10.92	9.08	0.000051	14.71	6.94	0.45
GH165	10.77	12.27	1	14.10	12.20	0.017	17.96	12.96	1
GH13_28	42.82	39.23	1	48.48	46.50	0.0000011	57.66	46.34	1
GH113	11.24	12.06	0.99	12.63	10.65	0.016	8.99	13.77	0.87
GH140	16.76	16.89	0.48	22.99	20.93	0.000027	30.66	16.00	0.48
GH143	9.18	8.89	0.73	14.16	11.87	0.000098	17.43	9.27	0.7
GH138	6.18	5.97	0.85	11.03	8.44	0.0000058	13.72	6.86	0.12
GT89	9.58	10.56	1	11.53	8.80	0.0016	7.86	9.68	0.13
GH43_32	7.18	6.26	1	8.28	4.94	0.0026	7.58	6.86	1
GH142	18.02	17.61	0.14	24.90	21.30	0.000045	26.63	22.31	0.97
GH141	51.01	47.59	0.43	61.54	57.60	3.3E-08	75.66	55.10	0.58
GH130	193.81	196.21	0.51	191.14	186.44	0.035	211.85	199.96	1
CBM9	17.39	17.50	1	24.10	18.60	0.005	21.59	22.06	1
GH43_22	14.53	15.35	1	18.74	13.24	0.0000014	13.44	16.04	0.096
GH13_31	70.27	83.32	1	78.28	72.51	0.02	67.14	67.04	0.27
GT76	33.32	31.86	0.52	38.58	28.01	0.0058	56.12	60.82	0.35
GH4	44.45	48.01	1	57.95	44.46	0.041	39.95	58.26	1

GT28	556.00	561.17	1	561.09	545.11	0.037	567.12	574.63	1
GT39	71.58	65.09	0.32	76.44	59.45	0.031	99.86	112.97	0.12
CBM34	43.57	48.14	0.17	59.42	40.79	0.0032	39.88	50.28	0.21
GH18	143.94	155.53	0.65	166.55	142.32	0.000019	123.34	166.95	0.78
GT35	503.17	491.44	0.12	507.87	481.34	0.0000038	503.26	515.72	0.54
GH25	485.81	476.07	0.14	477.55	447.15	0.041	433.10	474.80	1
GH13_39	164.75	173.90	0.062	199.71	146.87	0.0000069	132.23	206.28	0.34
CBM48	169.38	175.09	0.079	208.30	151.75	0.000019	130.12	207.19	1
GH13_9	221.88	232.19	0.079	260.91	202.77	0.00000073	179.80	263.02	0.69
CE4	841.50	847.27	0.27	928.68	804.25	0.0016	758.12	967.25	0.27

Supplementary table 8. Ingredients and nutritional composition of sow diets

	Diet for sows in different states	
	**Gestation**	**Lactation**
**Ingredients (%)**		
Corn	60	59
Soybean meal, dehulled	17.6	20.7
Wheat bran	18	8
Extruded soybean	0	8
98.5%L-Lys.Hcl	0.32	0.27
DL-Met	0.08	0.05
L-Thr	0.1	0.08
Limestone	1.1	1.1
Ca(HCO3)2	1.4	1.4
NaCl	0.4	0.4
Premix	1	1
**Nutritional composition**		
Digestible energy (MJ/Kg)	13.08	13.87
Metabolic energy (MJ/Kg)	12.66	13.42
Crude protein (%)	15.51	18.2
Crude fiber (%)	3.68	3.49
Lysine (%)	1.02	1.2
Methionine + Cysteine (%)	0.62	0.67
Threonine (%)	0.67	0.77
Ca (%)	0.83	0.86
Available phosphorus (%)	0.3	0.29
Total phosphorus	0.59	0.56
